# The incorporation of Mg^2+^ ions into aragonite during biomineralization: Implications for the dolomitization of aragonite

**DOI:** 10.3389/fmicb.2023.1078430

**Published:** 2023-01-26

**Authors:** Zuozhen Han, Ruirui Meng, Hui Zhao, Xiao Gao, Yanyang Zhao, Yu Han, Fang Liu, Maurice E. Tucker, Jiarong Deng, Huaxiao Yan

**Affiliations:** ^1^Shandong Provincial Key Laboratory of Depositional Mineralization and Sedimentary Minerals, College of Earth Science and Engineering, College of Chemical and Biological Engineering, Shandong University of Science and Technology, Qingdao, China; ^2^Laboratory for Marine Mineral Resources, Center for Isotope Geochemistry and Geochronology, Qingdao National Laboratory for Marine Science and Technology, Qingdao, China; ^3^School of Geosciences, China University of Petroleum, Qingdao, China; ^4^College of Chemical Engineering, China University of Petroleum, Qingdao, China; ^5^State Key Laboratory of Petroleum Pollution Control, Beijing, China; ^6^School of Earth Sciences, University of Bristol, Bristol, United Kingdom; ^7^Cabot Institute, University of Bristol, Bristol, United Kingdom

**Keywords:** dolomitization, molecular mechanism, Mg**^2+^** incorporation, acidic biomolecules, biotic aragonite

## Abstract

Bacteria can facilitate the increase of Mg^2+^ content in biotic aragonite, but the molecular mechanisms of the incorporation of Mg^2+^ ion into aragonite facilitated by bacteria are still unclear and the dolomitization of aragonite grains is rarely reported. In our laboratory experiments, the content of Mg^2+^ ions in biotic aragonite is higher than that in inorganically-precipitated aragonite and we hypothesize that the higher Mg content may enhance the subsequent dolomitization of aragonite. In this study, biotic aragonite was induced by *Bacillus licheniformis* Y_1_ at different Mg/Ca molar ratios. XRD data show that only aragonite was precipitated in the media with Mg/Ca molar ratios at 6, 9, and 12 after culturing for 25 days. The EDS and atomic absorption results show that the content of Mg^2+^ ions in biotic aragonite increased with rising Mg/Ca molar ratios. In addition, our analyses show that the EPS from the bacteria and the organics extracted from the interior of the biotic aragonite contain the same biomolecules, including Ala, Gly, Glu and hexadecanoic acid. The content of Mg^2+^ ions in the aragonite precipitates mediated by biomolecules is significantly higher than that in inorganically-precipitated aragonite. Additionally, compared with Ala and Gly, the increase of the Mg^2+^ ions content in aragonite promoted by Glu and hexadecanoic acid is more significant. The DFT (density functional theory) calculations reveal that the energy needed for Mg^2+^ ion incorporation into aragonite mediated by Glu, hexadecanoic acid, Gly and Ala increased gradually, but was lower than that without acidic biomolecules. The experiments also show that the Mg^2+^ ion content in the aragonite significantly increased with the increasing concentration of biomolecules. In a medium with high Mg^2+^ concentration and with bacteria, after 2 months, micron-sized dolomite rhombs were precipitated on the surfaces of the aragonite particles. This study may provide new insights into the important role played by biomolecules in the incorporation of the Mg^2+^ ions into aragonite. Moreover, these experiments may contribute towards our understanding of the dolomitization of aragonite in the presence of bacteria.

## 1. Introduction

Aragonite is an abundant carbonate mineral in modern shallow-marine sediments and was originally present in many Phanerozoic limestones (Gischler et al., 2013; Hasim and Kaczmarek, 2019, 2021). The precipitation of aragonite and hence abundance through time, however, does vary reflecting changes in the chemical composition of seawater, notably the Mg/Ca ratio, as well as the evolution and demise of skeletal organisms in their biomineralization (Sandberg, 1975, 1983; Balthasar and Cusack, 2015; Sun et al., 2015; Pederson et al., 2019; Hasim and Kaczmarek, 2021). The variation in seawater chemistry and hence aragonite vs. calcite precipitation is mostly controlled by geotectonic processes an rates of seafloor spreading. Aragonite is a metastable mineral and in time is usually replaced by the more stable calcite, particularly through contact with meteoric water [reviewed in Tucker and Jones (2023)]. The role played by bacteria in the biomineralization of aragonite has been studied in both natural environments and in laboratory experiments (Lian et al., 2006; Rodriguez-Navarro et al., 2007; Han et al., 2017; Perri et al., 2018; Yan et al., 2021). In modern microbial mats from Qatar, Perri et al. (2018) showed that aragonite precipitated together with low-Mg calcite and other carbonate minerals around bacteria and within extracellular polymeric substances (EPS). Stanley and Hardie (1998) suggested that some particular algae could preferentially promote the formation of aragonite. Needle-like aragonite is induced by the codiacean alga *Penicillus capitatus* (Ries, 2005), with organic compounds therein acting as the template (Borowitzka, 1984).

Significant efforts have been made to understand the biomineralization mechanism of aragonite induced by bacteria and algae (Ries, 2005; Obst et al., 2009; Balthasar and Cusack, 2015). It has been reported that the acidic amino acids, such as aspartic acid (Asp) and glutamic acid (Glu) in EPS, are able to adsorb Ca^2+^ ions, leading to the induction of aragonite (Kawaguchi and Decho, 2002). This result is also supported by the precipitation of aragonite under the action of soluble EPS extracted from *Shewanella piezotolerans* WP3 (Li et al., 2019). Han et al. (2017) suggested that pH and carbonate alkalinity were increased by bacterial metabolism, such as the production of NH_3_ (Han et al., 2017, 2018, 2019) and carbonic anhydrase (CA) (Zhuang et al., 2018). In these alkaline conditions, the negatively-charged EPS around bacteria can absorb Ca^2+^ and Mg^2+^ ions to create a locally supersaturated microenvironment, facilitating the nucleation of aragonite.

In the precipitation of aragonite, bacteria can also affect the proportion of Mg^2+^ in aragonite. As reported, the changes of Mg/Ca ratio in seawater can control the oscillations of the CaCO_3_ polymorph (Balthasar and Cusack, 2015) and high Mg/Ca ratios are favorable for the formation of aragonite (Folk, 1974; Berner, 1975; Sandberg, 1975; Morse et al., 1997; Ries et al., 2008; De Choudens-Sánchez and González, 2009; Balthasar and Cusack, 2015; Son et al., 2020). However, during this process, only a small amount of Mg^2+^ is actually incorporated into the aragonite due to the orthorhombic structure of aragonite (Sun et al., 2015; Wassenburg et al., 2016; Yao et al., 2019). In terms of aragonite crystal structure, nine O atoms surround one Ca atom, forming the nine Ca-O bonds, meaning that if a Mg^2+^ ion is to replace a Ca^2+^ ion, nine Ca-O bonds within the aragonite crystal need to be broken. This indicates that high energy is required to replace Ca^2+^ ions with Mg^2+^ ions in aragonite (Zhou et al., 2004; Sun et al., 2015; Yao et al., 2019). However, high contents of Mg^2+^ ions have been reported for organically-induced aragonite, quite different from inorganically-precipitated crystals (Finch and Allison, 2008; Holcomb et al., 2009; Gaetani et al., 2011). Yao et al. (2019) demonstrated that the precipitation of aragonite mediated by sodium alginate (SA) and carboxylation chitosan (CC) led to higher Mg^2+^ contents than those in inorganically-precipitated aragonite, and the Mg^2+^ content increased with increasing biomolecule concentrations. In addition, the Mg^2+^ content of aragonite mediated by SA is different from that mediated by CC, indicating an influence of the organic material itself on the incorporation of Mg^2+^ ions into aragonite (Yao et al., 2019). However, the content of Mg^2+^ in biotic aragonite and the molecular mechanisms that promote the incorporation of Mg^2+^ into biotic aragonite during biomineralization process have rarely been systematically studied. In addition, the transformation of dolomite from aragonite under normal temperatures and pressures is much more difficult to perform and has been rarely explored. We speculate that in the process of aragonite biomineralization, with respect to the increase of Mg^2+^ content, bacteria may be able to facilitate the dolomitization of aragonite, and this is one of the objectives with our experiments.

In this study, the precipitation of biotic aragonite was induced by *Bacillus licheniformis* Y_1_ bacteria at different Mg/Ca molar ratios. To analyze the chemical characteristics of the aqueous media, the concentrations of HCO_3_^–^, CO_3_^2–^, Ca^2+^ and Mg^2+^ ions in the culture media were determined periodically, and from these the saturation index (SI) of aragonite was calculated. The content of Mg^2+^ ions in the biotic aragonite precipitates was determined with atomic absorption spectroscopy (AAS). In order to explore the incorporation mechanism of Mg^2+^ into biotic aragonite affected by bacteria, the amino acids and volatile biomolecules within the EPS and biotic aragonite were analyzed. Alanine (Ala), glycine (Gly), Glu and hexadecanoic acid (HA) detected from the EPS and biotic aragonite were used as the template to study the influence of biomolecules on the incorporation of Mg^2+^ into biotic aragonite. Density-functional theory (DFT) calculations were used to determine the difference in energy needed for the incorporation of Mg^2+^ into aragonite in the absence and presence of biomolecules. Additionally, the dolomitization of aragonite was also studied in Mg^2+^-rich solutions. This study may help us to understand the important role played by biomolecules in Mg^2+^ incorporation into biotic aragonite and hence the influence of bacteria on the dolomitization of aragonite.

## 2. Materials and methods

### 2.1. Medium components, identification and morphology of the *Bacillus licheniformis* Y_1_ strain

The liquid medium containing the following components was prepared: beef extract 5 g/L, tryptone 10 g/L, and NaCl 10 g/L, and pH was adjusted to 7.0 using hydrochloric acid (HCl, 1 mol/L) and sodium hydroxide (NaOH, 1 mol/L). The medium was sterilized in an autoclave, and then cooled to room temperature. The solid medium is based on the liquid medium with an additional 22 g/L agar powder.

The *B. licheniformis* Y_1_ strain was preserved in the Geomicrobiology Lab at Shandong University of Science and Technology (Qingdao, China). The Y_1_ bacteria stored in the refrigerator at −20°C were taken out and diluted 1,000 times with sterile distilled water. The diluted bacteria solution was daubed on the surface of the solid medium and cultured in a constant temperature incubator (DNP-9162, China) at 37°C. After being cultured for 24 h, single colonies could be observed.

Some of the pure bacteria were sent to Bioengineering Co., Ltd. (Shanghai, China) for 16S rDNA sequencing. The 16S rDNA sequences obtained were uploaded to the GenBank/NCBI database and an accession number was obtained. The phylogenetic tree of the Y_1_ strain was constructed by the neighbor-joining method with MEGA software (version 3.1). The morphology of the bacterium Y_1_ was recorded with a scanning electron microscope (SEM, FEI Quanta 200, USA).

### 2.2. Biochemical characteristics of Y_1_ bacteria

One single colony on the surface of the solid medium in section “2.1. Medium components, identification and morphology of the Bacillus licheniformis Y_1_ strain” was selected out and inoculated into the liquid culture medium, cultured in a constant temperature shaker (TS-1102C, China) at 37°C for 24 h. The bacterial concentration was measured with a spectrophotometer (722 s, China) at 600 nm. When the bacterial concentration reached 7.2 × 10^8^ cfu/mL or so, the preparation of bacterial suspension was finished.

The sterilized liquid medium inoculated with the bacterial suspension (OD_600_ = 0.9) at an inoculation ratio of 1% (v:v) was set as the experimental group and the same volume of sterilized distilled water was added in the uninoculated control group. During the whole period of bacterial growth, the cell concentration was measured by a spectrophotometer at 600 nm, synchronously, pH values in the experimental and control groups were measured with a pH meter (PHS-3, China).

### 2.3. Biomineralization experiments

The medium used to induce the biominerlization of aragonite contained the following components: beef extract 5 g/L, tryptone 10 g/L, NaCl 10 g/L, Ca^2+^ 0.01 mol/L, and Mg^2+^ ions (0, 0.03, 0.06, 0.09, and 0.12 mol/L). In the biomineralization medium, magnesium chloride hexahydrate (MgCl_2⋅_6H_2_O) and calcium chloride (CaCl_2_) were used to adjust Mg/Ca molar ratios to 0, 3, 6, 9, and 12, respectively. Each separate biomineralization medium contained one kind of Mg/Ca ratio. The pH values of all the media were adjusted to 7.0 using hydrochloric acid (HCl, 1 mol/L) and sodium hydroxide (NaOH, 1 mol/L). All the media were sterilized in an autoclave, and then cooled to room temperature. The control and experimental groups were also set according to the detailed step 2.2. Each Mg/Ca ratio was triplicated in both the control and experimental groups. All the flasks were incubated at 37°C with a rotation speed of 130 rpm.

### 2.4. Determination of HCO_3_^–^, CO_3_^2–^, Ca^2+^, Mg^2+^, pH, and saturation index in the biomineralization process

During the experimental period, 1 mL of suspension at different Mg/Ca molar ratios was taken out periodically, diluted with distilled water and filtered through a 0.22 μm pore-sized membrane. Then, Ca^2+^ and Mg^2+^ concentrations in the filtrate were analyzed by atomic absorption spectroscopy (AAS, TAS-986F, China). The determination of concentrations of HCO_3_*^–^* and CO_3_^2^*^–^* was performed according to our previous methods (Zhuang et al., 2018). pH was measured with a pH meter. The saturation index (SI) of carbonate minerals in the media was calculated with the software PHREEQC (Parkhurst and Appelo, 2013). The significance in HCO_3_*^–^*, CO_3_^2^*^–^*, Ca^2+^, Mg^2+^, pH, and saturation index between various culture media was analyzed with IBM SPSS statistics software.

### 2.5. Characterization of the bio-minerals

On the 14th and 25th day, the bio-minerals were harvested and washed 3 times with distilled water and anhydrous ethanol, respectively, to remove the medium and bacterial debris. After drying, the bio-minerals were analyzed by X-ray powder diffraction (XRD, D/Max/2500PC, Japan) with a scanning speed of 8°/min, a step size of 0.02° and 2θ angle range 10–80° to determine the mineral phases. The Rietveld refinement of the bio-minerals was performed by Material Studio 7.0 software. Morphologies and elemental compositions of the bio-minerals were analyzed with SEM and energy-dispersive X-ray spectroscopy (EDS). The thoroughly cleaned bio-minerals were hydrolyzed by hydrochloric acid and the hydrolysates were also analyzed with Fourier transform infrared spectroscopy (FTIR). The bio-minerals were also labeled with Cy5 NHS ester (Xian Kaixin biotechnology Co., Ltd.) and observed with a laser scanning confocal microscope (LSCM, LSM700, Germany) at an excitation wavelength of 630 nm.

### 2.6. Analyses of ultrathin slices of bacteria

The bacteria were collected from the culture media to prepare ultrathin slices, and the detailed steps were according to Han et al. (2017). The ultrathin slices were analyzed by high-resolution transmission electron microscopy (HRTEM, Hitachi H-7650, Japan), selected area electron diffraction (SAED), and mapping with a scanning transmission electron microscope (STEM, Tecnai G2 F20, America), accompanied with EDS.

### 2.7. Determination of components in EPS

Bacterial EPS were extracted with the heating method (Zhuang et al., 2018), dried in a lyophilizer (FD-1A-50, China) at −58°C under vacuum conditions. EPS powder was sent to Jiangsu Coastal Chemical Analysis & Technological Service Ltd to analyze the amino acids in EPS by an L-8900 amino acid analyzer (Hitachi, Japan). EPS powder was also analyzed by FTIR (Nicolet 380, America) with a potassium bromide (KBr) disk method at a scope of infrared spectroscopy range 4,000–400 cm^–1^. In order to analyze the volatile organic compounds (VOC) in EPS, the EPS powder was dissolved with anhydrous alcohol; the supernatant was filtered by a membrane with a pore size of 0.22 μm, and the water in the filtrate was sucked away by dried sodium sulfate (Na_2_SO_4_). The solution was then analyzed by gas chromatography-mass spectrometry (GC-MS, 7890/5975C, America) with HP-5MS (5% Phenyl methyl silox) as the column, and helium as the carrier gas. The temperature was kept at 60°C for 0.25 min, then increased from 60°C to 300°C at a rate of 10°C/min, and finally kept at 300°C for 6 min.

### 2.8. VOC and amino acids within biotic aragonite

The biotic aragonite was soaked in sodium hypochlorite (NaClO) until no air bubbles were produced, and washed with deionized water and anhydrous ethanol three times (10 min each time), then soaked in anhydrous ethanol for 1 day. Finally the supernatant was taken out to be analyzed by GC-MS. To test whether the supernatant contained VOC, anhydrous ethanol as the control was also detected with GC-MS at the same time. If no excrescent VOC were present in the supernatant, VOC were fully removed from the surface of the biotic aragonite. The clean biotic aragonite was dissolved with hydrochloric acid for 1 day and then diluted with distilled water and filtered with a 0.22 μm pore-sized membrane. The obtained filtrates were dried into a powder at −58°C under vacuum. One part of the powder was tested by an amino acid analyzer, and the other part was dissolved in anhydrous ethanol and analyzed with GC-MS.

### 2.9. Mg^2+^ content in the biotic aragonite

Mg^2+^ contents of the biotic aragonites cultured at Mg/Ca ratios of 6, 9, and 12 were analyzed according to the following steps: the biotic aragonite was washed with distilled water until Mg^2+^ concentration in the supernatant was zero; after being dried and weighed, aragonite was dissolved in hydrochloric acid; then, the solution was diluted and filtered with a 0.22 μm pore-sized membrane; the Mg^2+^ concentration in the filtrate was measured by the AAS method.

### 2.10. Synthesis of organically-precipitated and inorganically-precipitated aragonite

The Glu, Gly, and Ala solutions at various concentrations (0.05, 0.10, and 0.15 g/L) and the saturated HA solution were prepared with a volume at 150 ml respectively. CaCl_2_ and MgCl_2_⋅6H_2_O were added to make the Ca^2+^ concentration at 0.01 M and a Mg/Ca molar ratio at 6. The above organic solutions containing a Mg/Ca ratio of 6 were heated until the temperature reached 37°C; 10 mL of sodium carbonate (Na_2_CO_3_) solution (0.02 mol/L) was dripped into the heated organic solutions respectively. After that, the mixture was stirred by magnetic stirrers (78-1, China) with a speed of 1,000 rpm, and cultured at 37°C (± 1) for 2 h. Then, the solutions were moved in a constant temperature incubator and cultured at 37°C (± 1) for 2 days to harvest more organically-precipitated minerals. The inorganically-precipitated minerals were prepared according to the above mentioned method, and the only difference was that the organic molecules were not added. The harvested organically-precipitated minerals and inorganically-precipitated minerals were analyzed with XRD and SEM after washing with distilled water and drying.

### 2.11. Mg^2+^ content in the organically-precipitated and inorganically-precipitated aragonite

The organically-precipitated and inorganically-precipitated aragonites were all thoroughly washed with distilled water. All the following experimental steps used to measure Mg^2+^ content were same as those mentioned in section “2.9. Mg^2+^ content in the biotic aragonite.”

### 2.12. Interplanar spacing analyses of three kinds of aragonite with HRTEM and SAED

Aragonite precipitates formed under the conditions of physical and chemical factors, Glu and bacteria were ground in an agate mortar, respectively, and then the three groups of aragonite powder were sieved with a 200- mesh sieve; finally, the sieved powder was suspended with anhydrous ethanol. The suspension was dripped onto the surface of a copper mesh containing an ultra-thin carbon film, and, after drying, analyzed with HRTEM and SAED. Digital Micrograph software was used to calculate the interplanar spacing of the three kinds of aragonite to further explore whether the interplanar spacing of some *hkl* planes from the aragonite induced by bacteria decreased due to the incorporation of Mg^2+^ ions.

### 2.13. DFT calculation

The Mg^2+^ ions embedded in the aragonite (111) crystal plane were studied by the Dmol3 module. The first-principles DFT calculations were carried out using the Dmol3 module in Materials Studio. The electron exchange–functional was calculated using Perdew-Burke–Ernzerhof (PBE) described by generalized gradient approximation (GGA). An all electron double numerical atomic orbital augmented by Double Numerical plus polarization (DNP) is used as the basis set and DFT semicore pseudopotentials (DSPPs) were used for the interactions between the ion core and the valence electrons; the Nudged Elastic Band (NEB) method was used to study the diffusion of Mg^2+^ ions. The convergence criteria in total energy, maximum force, and maximum displacement were set at 10^–5^ Hartree, 0.002 Hartree/Å, and 0.005 Å, respectively. The electronic self-consistent field (SCF) tolerance was set at 10^–6^ Hartree.

### 2.14. HRTEM, EDS and elemental analyses of minerals after the dolomitization of bio-aragonite

After the aragonite induced by bacteria was analyzed by SEM-EDS, the atom ratio of Mg: Ca in some aragonite was found to be almost 1:1. Such aragonite like this (Mg/Ca = 1:1) would be recycled from the up-holder, and ground with an agate mortar. The powder was suspended with anhydrous ethanol, then the suspension was dripped onto the surface of copper mesh containing a ultra-thin carbon film; after drying, it was analyzed with HRTEM, EDS and elemental analyses. Digital Micrograph software was used to calculate the interplanar spacing.

## 3. Results

### 3.1. Identification and characterization of the *Bacillus licheniformis* Y_1_ strain

#### 3.1.1. Molecular identification, morphology and ammonia test of the *Bacillus licheniformis* Y_1_ strain

The 16S rDNA sequence of the Y_1_ strain (accession number MF808044.1) was 1,387 bp long and owned 99.93 % homology with those of 60 strains of *B. licheniformis* in Genbank. As seen from the phylogenetic tree (Supplementary Figure 1), strain Y_1_ was most closely related with the *B. licheniformis* family. Thus, strain Y_1_ was identified as *B. licheniformis*, which was about 1.1 μm long and 0.7 μm wide (Supplementary Figure 2A). It can be clearly seen that the cell is wrapped in sticky EPS, especially the two ends of the cell (Supplementary Figure 2B). The ammonia test proved that *B. licheniformis* Y_1_ could release ammonia, because the experimental group presented a brown color and the control group turned into yellow after Nessler’s reagent was added (Supplementary Figures 2B, C).

#### 3.1.2. Growth curve and pH changes of *B. licheniformis* Y_1_

The growth process of *B. licheniformis* Y_1_ was divided into four stages: adaptive stage (0–4 h), logarithmic stage (4–14 h), stationary stage (14–20 h) and decline stage (20 h–end) (Supplementary Figure 3). As for pH changes, the control group remained almost unchanged around 7.0, whereas the experimental group slightly decreased at the beginning, then sharply increased, and lastly kept stable at 8.8 or so (Supplementary Figure 3).

#### 3.1.3. Changes of pH, HCO_3_^–^, CO_3_^2–^, Ca^2+^ and Mg^2+^ ion concentrations and SI during biomineralization

**FIGURE 1:**
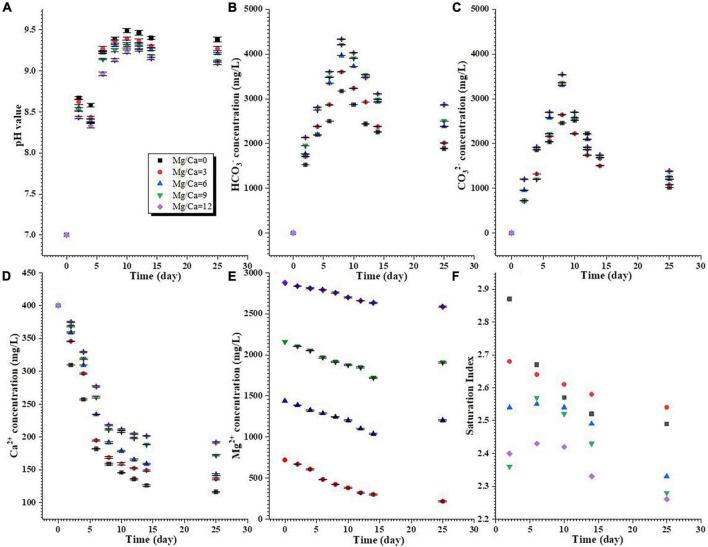
Changes in chemical conditions of the aqueous media during the process of biomineralization. **(A)** pH value; **(B,C)** HCO_3_^–^ and CO_3_^2–^ concentrations; **(D,E)** Ca^2+^ and Mg^2+^ concentrations; **(F)** calculated saturation index of aragonite.

During the biomineralization process, the pH in the media with different Mg/Ca molar ratios had increased from 7.0 to over 9.0 by day 25 (Figure 1A). The concentrations of HCO_3_^–^ and CO_3_^2–^ increased from the beginning of the experiment to the 8th day, and then decreased from the 8th to 25th day (Figures 1B, C). In the whole experimental period, the concentrations of HCO_3_^–^ were higher than those of CO_3_^2–^ (Figures 1B, C). The concentrations of Ca^2+^ were sharply decreased from the 0th to the 8th day, and then the trend changed more slowly (Figure 1D). The concentrations of Mg^2+^ at a Mg/Ca molar ratio of 3 decreased more quickly than those at other Mg/Ca molar ratios (Figure 1E). As shown in Figure 1F and Supplementary Figure 4, the culture media were supersaturated with respect to aragonite and calcite during the precipitation experiments. The different phases of carbonate minerals may be related to the selection of bacteria and Mg/Ca molar ratios. There is an extremely significant difference in change of pH, HCO_3_^–^, CO_3_^2–^, Ca^2+^, and Mg^2+^ ion concentrations and SI between different Mg/Ca ratios at the same time (Supplementary Tables 1–6).

### 3.2. Biogenesis of minerals

#### 3.2.1. Phases of the bio-minerals

**FIGURE 2:**
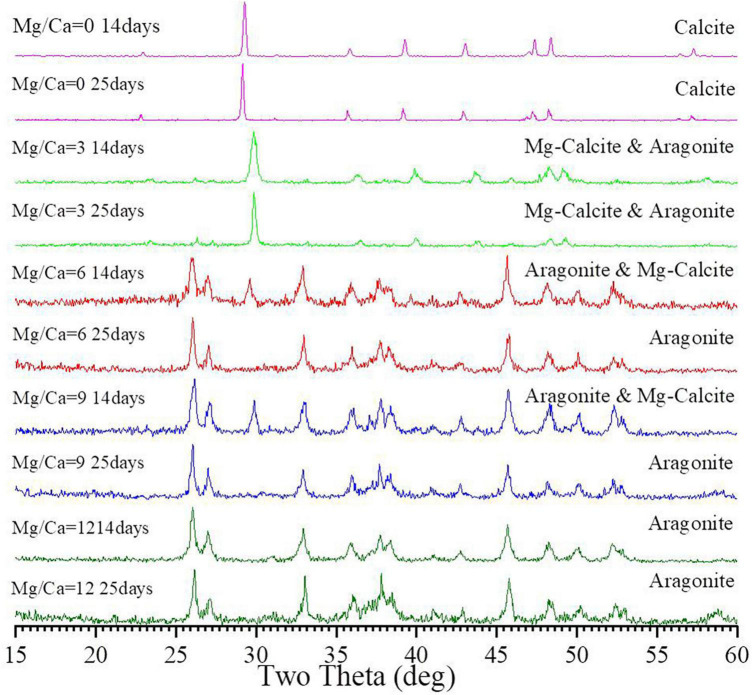
X-ray powder diffraction (XRD) analyses of the bio-minerals induced by *Bacillus licheniformis* Y_1_ at different Mg/Ca molar ratios after 14 and 25 days of culture.

Biotic calcite was formed at a Mg/Ca molar ratio of 0 both on 14th and 25th day (Figure 2), which was further confirmed by Rietveld refinement (Supplementary Figures 5A, B). Mg-calcite and aragonite were obtained at a Mg/Ca molar ratio of 3 (Figure 2). Compared to the 14th day, the mass ratio of Mg-calcite increased from 83.9% (Supplementary Figure 5C) to 96.4% (Supplementary Figure 5D) after culture for 25 days. At Mg/Ca molar ratios of 6 and 9, the precipitates were also determined as Mg-calcite and aragonite. However, with the increase in culture time, the mass ratio of aragonite within the precipitates increased from 95.5 % (Supplementary Figure 5E) and 97.3 % (Supplementary Figure 5G) to 100 % (Supplementary Figures 5F, H), respectively. Both detected bio-minerals were only aragonite at a Mg/Ca molar ratio of 12, no matter whether the 14th or 25th day (Figure 2), and this was confirmed by Rietveld refinement (Supplementary Figures 5I, J). XRD results also show that the interplanar spacing of aragonite *hkl* plane (111) (PDF#71-2396) decreased with increasing Mg^2+^ ion concentration from Mg/Ca ratio of 6 through 9 to 12 (Supplementary Table 7). It can be seen from Supplementary Table 7 that with the increasing Mg^2+^ ion concentrations, the interplanar spacing of the aragonite *hkl* plane (111) decreases from 3.427 Å through 3.419 Å to 3.411 Å on 14th day and from 3.421 Å through 3.416 Å to 3.404 Å on 25th day.

#### 3.2.2. Morphology of the bio-minerals

**FIGURE 3:**
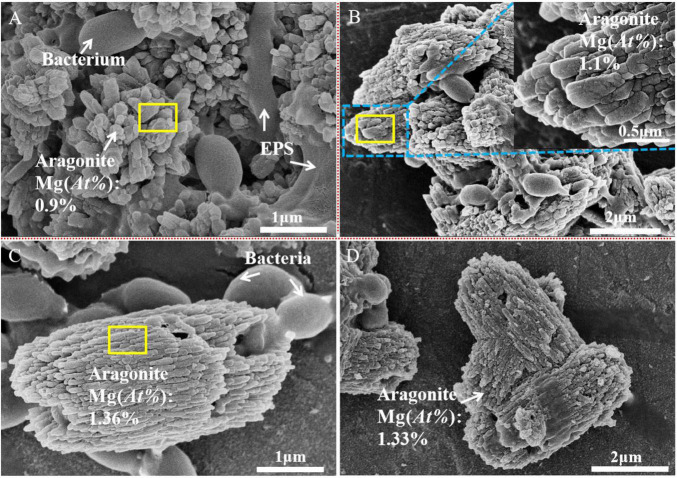
Scanning electronic microscope images of the biotic aragontie induced by *Bacillus licheniformis* Y_1_ at different Mg/Ca ratios. **(A)** Biotic aragonite formed at a Mg/Ca molar ratios of 6; **(B)** biotic aragontie formed at a Mg/Ca molar ratio of 9; and **(C,D)** biotic aragonite formed at a Mg/Ca molar ratio of 12.

The morphologies of the bio-minerals induced by *B. licheniformis* Y_1_ became more diverse with increasing Mg^2+^ concentrations. Biotic calcite shows rhombic, elongate and X-shaped morphologies with rough surfaces (Supplementary Figures 6A–C). EDS results show that C, O, Ca, Al, and P elements are present in the calcite (Supplementary Figure 7A). C, O and Ca mainly come from the biotic calcite; the element Al is from the sample holder, and the element P is probably from metabolites of *B. licheniformis* Y_1_ and/or the bacteria. In Supplementary Figures 6B, C, bacteria adhered to the calcite can be clearly observed. In the presence of Mg^2+^, the biotic Mg-calcite crystallites form aggregates. Cavities derived from the shedding of bacterial thallus can be clearly seen in the Mg-calcite (Supplementary Figures 6D–H). Similar to the Mg-calcite, the biotic aragonite precipitated in this experiment is also in the form of crystallite aggregates (Figures 3A–D). The bacteria thallus and EPS adhered to the biotic aragonite can be seen in the SEM images (Figures 3A–D). The EDS results show that as the Mg/Ca molar ratio increases, the Mg^2+^ content (At%) in the biotic aragonite increases from 0.9% to around 1.1% or 1.4% (Supplementary Figures 7C–E). Since EDS is a semi-quantitative analysis, the Mg^2+^ concentrations in the biotic aragonites were further determined with AAS.

#### 3.2.3. Organic functional groups in the biotic aragonite

The characteristic bands of aragonite are 1,490 cm^–1^, 1,475 cm^–1^, 1,082 cm^–1^, 856 cm^–1^, and 710 cm^–1^ (Yan et al., 2020). As shown in Supplementary Figure 8, the bands 1,483 cm^–1^, 1,081 cm^–1^, 856 cm^–1^, and 710 cm^–1^ prove the formation of aragonite at Mg/Ca ratios of 6, 9, and 12. In addition to these characteristic bands of biotic aragonite, some bands of organic functional groups are also present, such as the stretching vibration of C = O in aldehyde at 1,786 cm^–1^ (Supplementary Figure 8) (Worzakowska et al., 2019), the C = O stretching vibration of amide I at 1,640 cm^–1^ (Supplementary Figure 8) (Pavlou et al., 2018), the aromatic C-H bending vibration at 1,424 cm^–1^ (Supplementary Figure 8) (Wang et al., 2019), disaccharides at 1,116 cm^–1^ (Supplementary Figure 8) (Andrade et al., 2020), and the vibration of C-H in benzene at 800 cm^–1^ (Supplementary Figure 8) (Qin et al., 2010). In addition, the proteins present in the biotic aragonite at different Mg/Ca molar ratios have different secondary structures (Table 1). Structures like β-sheet (1,638 cm^–1^) are found in aragonite at ratios of 6 and 9, and β-sheet (1,640 cm^–1^ and 1,631 cm^–1^), 3_10_ helix (1,664 cm^–1^) and β-turn (1,672 cm^–1^) can be seen in aragonite precipitated at a Mg/Ca molar ratio of 12.

**TABLE 1:** Protein secondary structures in the biotic aragonite cultured for 25 days.

Mg/Ca molar ratio	Protein secondary structure (cm^–1^)			
	β-sheet	α-helix	3_10_ helix	β-turn
6	1,638	-	-	-
9	1,638	-	-	-
12	1,640, 1,631	-	1,664	1,672

**FIGURE 4:**
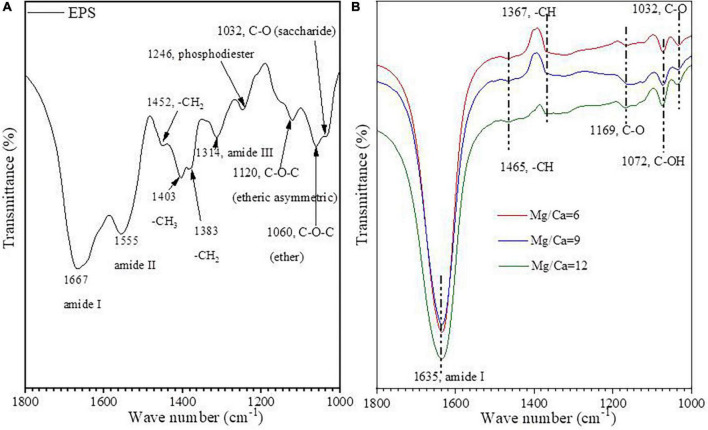
FTIR spectra of EPS **(A)** and hydrolysate of biotic aragonite hydrolysed by hydrochloric acid **(B)**.

The hydrolysate of biotic aragonite and the EPS of bacteria Y_1_ were further analyzed by FTIR. Some organic functional groups such as amide I (1,667 cm^–1^), amide II (1,555 cm^–1^), -CH_2_ (1,452 cm^–1^ and 1,383 cm^–1^), -CH_3_ (1,403 cm^–1^), amide III (1,314 cm^–1^), phosphodiester (1,246 cm^–1^), -C-O-C- (1,120 cm^–1^ and 1,060 cm^–1^) (Alasonati and Slaveykova, 2012), and -C-O of saccharide (1,032 cm^–1^) (Wang Y. Y. et al., 2013) were detected in the EPS (Figure 4A). The FTIR results from the hydrolysate show that amide I (1,635 cm^–1^), -C-H (1,465 cm^–1^ and 1,367 cm^–1^), -C-O-C- (1,169 cm^–1^), -C-OH (1,072 cm^–1^), -C-O (1,032 cm^–1^) (Wang Y. Y. et al., 2013) were present within the biotic aragonite (Figure 4B). Based on the fact that EPS can act as nucleation sites, it can be concluded that amide I and -C-O in the minerals may have come from EPS due to the fact that both EPS and the mineral hydrolysate have the same two organic functional groups. This conclusion suggests that certain organic molecules may regulate carbonate mineral nucleation and growth.

### 3.3. Extracellular biomineralization of *B. licheniformis* Y_1_ cells

When analyzing the morphology of bio-minerals, it was found that there were nanoscale polygonal crystals on the surface of bacteria and bacterial EPS (Supplementary Figure 9A). With the incubation time, the amount of nanoscale polygonal crystals increased (Supplementary Figure 9B) and finally a mineralized shell encasing the bacterium was formed (Supplementary Figure 9C). The areas marked with pink circles in Supplementary Figure 9A also show that the mineralized shell was forming and the mineralized shell was incomplete. To test whether EPS acted as the nucleation site, ultrathin slices were prepared and analyzed by HRTEM, EDS, SAED, and elemental mapping.

**FIGURE 5:**
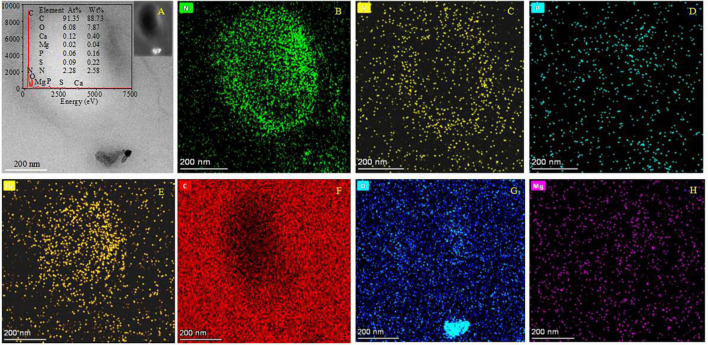
HRTEM and elemental mapping images of ultrathin slices of *B. licheniformis* Y_1_ cells. **(A)** The morphology of bacteria and the bio-minerals on the cell surface in a bright field. The left inset in panel **(A)** is the EDS image of the whole cell; the right inset in panel **(A)** is the morphology of bacteria and the bio-mineral on the cell surface in a dark field. **(B–H)** Indicating the mapping of elements N, P, S, Ca, C, O, and Mg.

**FIGURE 6:**
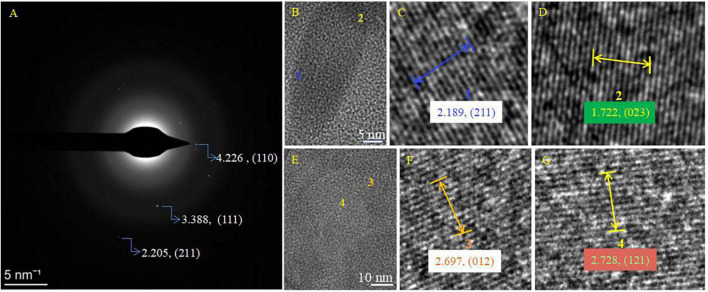
SAED and HRTEM images of the biominerals on the cell surface in Figure 5A. **(A)** SAED image; **(B–G)** HRTEM images. **(C,D)** HRTEM images of the selected area marked with 1 and 2 in panel **(B)**; **(F,G)** HRTEM images of the selected area marked with 3 and 4 in panel **(E)**. PDF card used: Aragonite PDF#71-2396.

It was found that the biomineralization of *B. licheniformis* Y_1_ cells does vary, depending on conditions. No minerals were formed on the cell outer surface when there were no metallic ions present in the medium (Supplementary Figure 10A). At the initial stage of biomineralization, occurring at different Mg/Ca ratios, extracellular bio-precipitation (Supplementary Figures 10B–D) can be observed through the formation of amorphous particles marked by yellow arrows in Supplementary Figure 10. In the later stages of biomineralization, nanometer-scale minerals formed on the cell surface and within the EPS (Figure 5A and Supplementary Figures 11A–D). EDS results show that some nanoscale minerals contain C, O, Ca, Cl, Cu and Os (Supplementary Figure 11E), whereas others contain Mg in addition to these elements (Supplementary Figure 11F). C is from the carbon membrane of the copper net, the bacterium, and the nanoscale minerals. O and Ca mainly come from the nanoscale minerals, Cl from the culture medium and Cu from the copper net. Os is due to the osmic acid that was used to prepare the ultrathin slices. Mg is mainly derived from the culture medium. These results prove that the nanoscale minerals on the cell surface and in the EPS are CaCO_3_, indicating that the EPS and/or cell surfaces have acted as nucleation sites. The diffraction spots in the SAED images (Supplementary Figures 11E, F, the original images of insets are in Supplementary Figure 12) reveal that the nanoscale minerals on the cell surface and in the EPS have weak crystalline structures. EDS result and elemental mapping of N, P, and S (Figures 5A–D) indicate that the landscape of a bacterium was depicted according to the morphology of *B. licheniformis* Y_1_ bacterium in Figure 5A. It can also be seen that Ca, C, O and Mg elements are also present inside the cell and EPS (Figures 5A, E–H), suggesting that Ca^2+^ and Mg^2+^ ions can be adsorbed on/in the EPS and also diffuse into the cell through the ion channels. The nanometer-scale minerals formed on the cell EPS (Figure 5A) were further analyzed by HRTEM and SAED. SAED results show that the calculated interplanar spacings are 2.205, 3.388, 4.226 Å, corresponding well to (211), (111), and (110) diffractions of aragonite (PDF#71-2396) (Figure 6A). HRTEM results show that the interplanar spacings of two regions marked with number 1 and 2 (Figure 6B) are 2.189 Å and 1.722 Å (Figures 6C, D), respectively matching well the two (211) and (023) diffractions of aragonite (PDF#71-2396) (Figures 6C, D). Moreover, two other areas marked with 3 and 4 (Figure 6E) were also analyzed and the results show that the calculated interplanar spacings (Å) are 2.697 and 2.728 (Figures 6F, G), consistent with (012) and (121) diffraction of aragonite (PDF#71-2396). Thus, the bio-mineral on/in the bacterial EPS is confirmed aragonite.

### 3.4. Amino acid components in EPS and in biotic minerals

**FIGURE 7:**
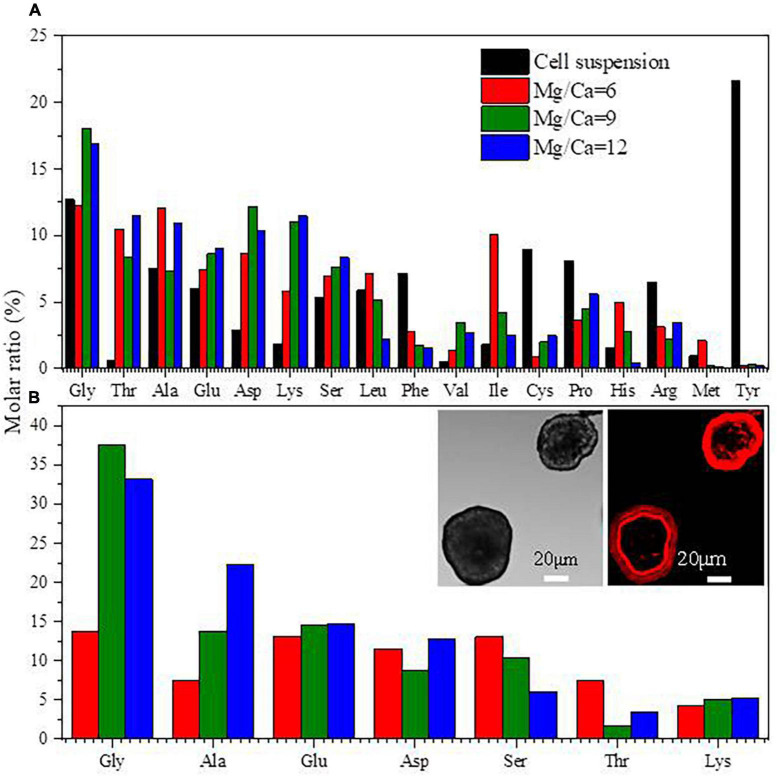
Amino acids in extracellular polymeric substances (EPS) **(A)** and amino acids in biotic aragonite **(B)**. The insets in panel **(B)** are the images of minerals labeled by CY5 NHS ester. The minerals in left inset are in visible light, and the right emitting the red fluorescence were at an excitation wavelength of 630 nm, revealing that the proteins comprising the amino acids are wrapping around the minerals.

Seventeen kinds of amino acid in total were detected in the EPS of *B. licheniformis* Y_1_ cells (Figure 7A). The contents of Gly, Ala and Glu in biotic aragonite and EPS were relatively higher in both than the other kinds of amino acid (Figure 7). The fluorescence of Cy5-NHS ester dyes reveals the existence of proteins in minerals (Bjerneld et al., 2015; Zhao et al., 2019). The left inset (Figure 7B) shows the morphology of minerals in visible light. The right one (Figure 7B) displays red fluorescence, which was emitted after the minerals were labeled with Cy5-NHS ester. Strikingly, it can be seen from the fluorescence image that proteins are wrapped around the minerals regularly in the shape of a ring. This phenomenon suggests that proteins can adsorb onto the mineral surfaces regularly or minerals grow directly under the regulation of proteins during the biomineralization process, which eventually facilitated the formation of different morphologies of CaCO_3_.

### 3.5. VOC in the EPS and biotic aragonite

**FIGURE 8:**
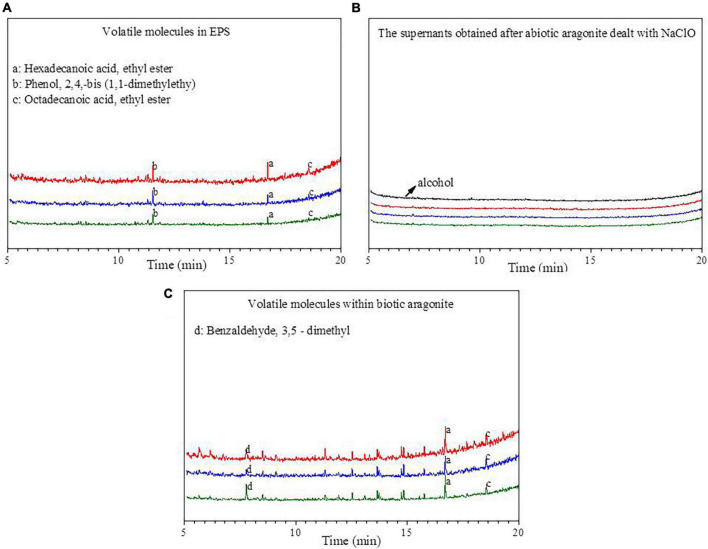
The spectra of GC-MS analyses. **(A)** Volatile molecules in extracellular polymeric substances (EPS); **(B)** the supernatants obtained after the biotic aragonite treated with NaClO and then re-suspended with alcohol; **(C)** volatile molecules within biotic aragonite. The red, blue and green lines represent the Mg/Ca molar ratios of 6, 9, and 12, respectively.

The organic molecules within the EPS and biotic aragonite were analyzed by GC-MS (Figure 8). The organic molecules in the EPS are mainly identified as: HA, ethyl ester; phenol, 2, 5-bis (1, 1–dimethylethyl) and octadecanoic acid, ethyl ester (Figure 8A). After the biotic aragonite was treated with NaClO and washed with deionized water, it was soaked in anhydrous ethanol and the supernatant was analyzed by GC-MS. The results show that the spectra of the supernatant are identical to those of anhydrous ethanol (Figure 8B), illustrating that the volatile biomolecules adsorbed onto the mineral surfaces had been removed. The volatile biomolecules within the biotic aragonite were also analyzed, and the results reveal that HA, ethyl ester and octadecanoic acid, ethyl ester are also present in the biotic aragonite (Figure 8C). Since anhydrous ethanol was selected to be the solvent, the organic matter obtained in this experiment should be the reaction product of the original substance with anhydrous ethanol. Therefore, the original substances of the products (a) HA, ethyl ester and (c) octadecanoic acid, ethyl ester shown in Figures 8A, C should be HA and octadecanoic acid. According to the GC-MS results, it can be inferred that HA and octadecanoic acid present in the EPS took part in the bio-precipitation of the biotic aragonite.

### 3.6. Mg content in the biotic aragonite

The contents of Mg^2+^ ions in the biotic aragonites formed at Mg/Ca molar ratios of 6, 9, and 12 on the 25th day were 3.8, 5.3, and 6.8 mg/g (Table 2), respectively. The significant differences (*p* < 0.01) in Mg^2+^ contents suggest that more Mg^2+^ ions can be incorporated into biotic aragonite with the increasing Mg^2+^ concentrations and the presence of *B. licheniformis* Y_1_.

**TABLE 2:** The changes of Mg content in biotic aragonite.

Mg/Ca molar ratios	Mean value (mg/g)
6	3.8**
9	5.3**
12	6.8**

**Significant difference (*p* < 0.01).

### 3.7. Biomimetic mineralization of aragonite mediated with various biomolecules

**FIGURE 9:**
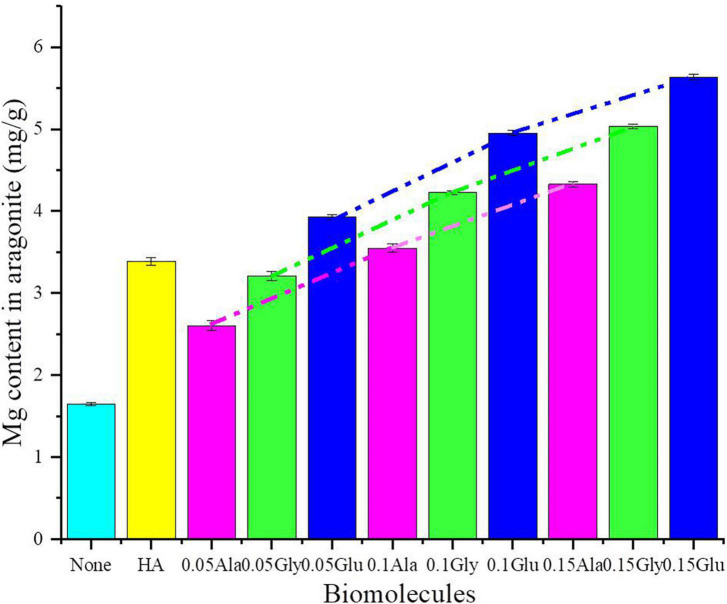
Mg content in aragonite precipitates mediated by various biomolecules.

Aragonite was successfully precipitated under the mediation of various concentrations of Glu, Gly, Ala, and HA at 37°C (± 1). The SEM images show that aragonite precipitated under all conditions formed aggregates of rod-like crystals (Supplementary Figure 13). However, the aragonite aggregates mediated by biomolecules were much looser with relatively round corners than the inorganically-precipitated aragonite (Supplementary Figure 13). As can be seen from Supplementary Figure 13, the influences of Glu on aragonite morphology were greater than the other biomolecules. As shown in Figure 9, although HA is slightly soluble in water, the Mg^2+^ content in aragonite mediated by HA was higher than the inorganically-precipitated aragonite and the aragonite precipitates mediated by 0.05 g/L Ala and Gly. Additionally, with increasing biomolecule concentrations (0.05, 0.10, and 0.15 g/L), the Mg^2+^ content in aragonite increases gradually. Generally, compared to other biomolecules, the incorporation of Mg^2+^ into aragonite facilitated by Glu was more effective. There was an extremely significant difference in Mg^2+^ content incorporated into the biotic aragonite mediated by different biomolecules (Supplementary Table 8).

### 3.8. SAED and HRTEM analyses of aragonite precipitates formed in the presence of physical and chemical conditions, Glu and bacteria

**FIGURE 10:**
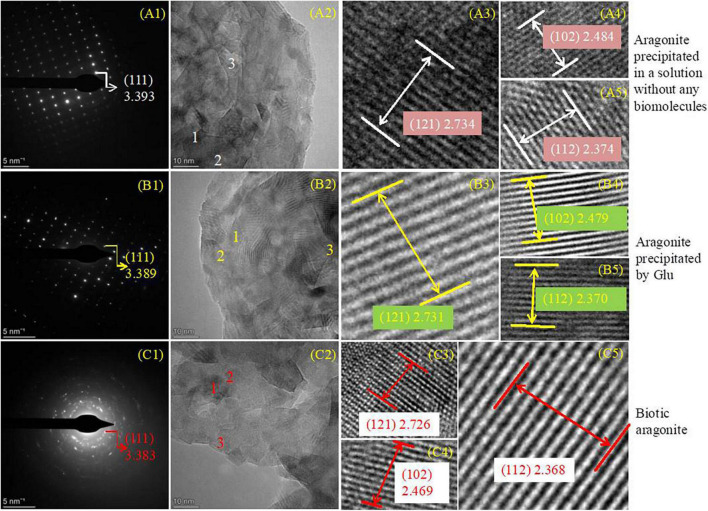
SAED and HRTEM analyses of aragonites precipitates. **(A1–C5)** Indicating aragonite formed in an inorganic solution, in the solution with the presence of Glu or bacteria, respectively. **(A1,B1,C1)** Are SAED images; **(A2–A5)**, **(B2–B5)**, and **(C2–C5)** are the HRTEM images.

In view of the fact that the interplanar spacing *d* (Å) of aragonite crystals would decrease when Mg^2+^ ions enter the aragonite lattice to replace Ca^2+^ ions, aragonite precipitates formed in the presence of physical and chemical conditions, Glu and bacteria were studied with SAED and HRTEM to further explore the change in the interplanar spacing of these three kinds of aragonite. The results show that the *hkl* planes (111), (121), (102), and (112) were obtained in the three kinds of aragonite (Figure 10, PDF#71-2396). The interplanar spacing *d* (Å) of aragonite crystal *hkl* plane (111) decreased from 3.393 through 3.389 to 3.383 (Figures 10A1–C1) after SAED analyses. Figures 10A2–C2 show the morphology of the three kinds of aragonite and three regions were selected to be further analyzed in each kind of aragonite. The interplanar spacing *d* (Å) of *hkl* plane (121) decreased from 2.734 through 2.731 to 2.726 (Figures 10A3–C3), that of *hkl* plane (102) decreased from 2.484 through 2.479 to 2.469 (Figures 10A4–C4), and that of *hkl* plane (112) decreased from 2.374 through 2.370 to 2.368 (Figures 10A5–C5). The interplanar spacing *d* values of *hkl* planes (111), (121), (102), and (112) decreased from the inorganically-precipitated aragonite through organically-precipitated aragonite in the presence of Glu to aragonite induced by bacteria. These results indicate that Mg^2+^ ions can more easily enter the aragonite lattice in biological systems. The difference in the reduction of the interplanar spacings may be related to the amount of incorporated Mg^2+^ ions in the aragonite.

### 3.9. Energy needed for the incorporation of Mg^2+^ into aragonite mediated by various biomolecules

**FIGURE 11:**
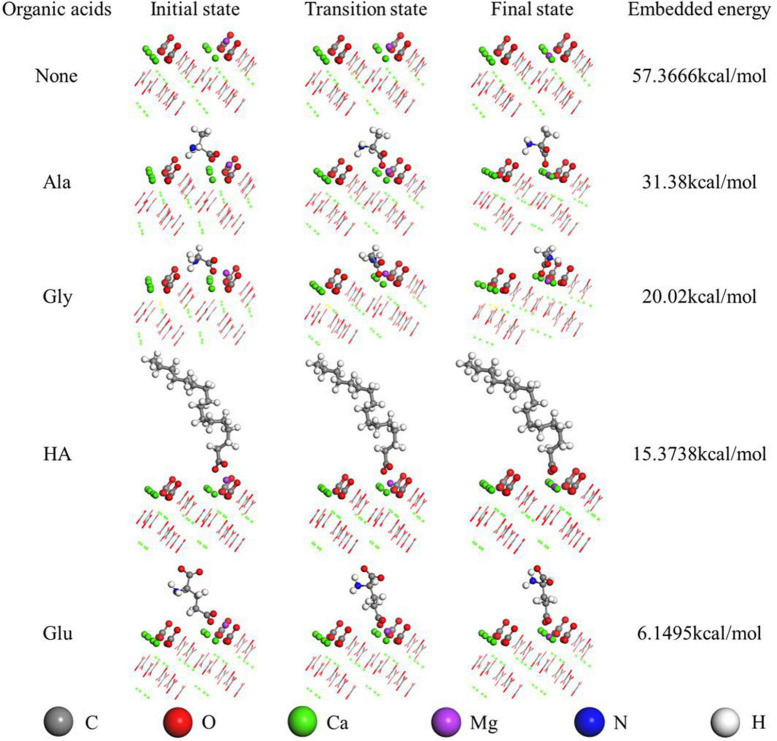
Mg^2+^ embedded in the aragonite (111) crystal face mediated by various organic acids studied by Dmol3 module. The first-principles density-functional theory calculations were carried out using the Dmol3 module in Materials Studio.

As for the organically-precipitated aragonite, the energy used for the Mg^2+^ ion incorporation into aragonite mediated by Glu, HA, Gly, and Ala is 6.15, 15.38, 20.02, and 31.38 kcal/mol (Figure 11), indicating that under the action of Glu, the energy required for Mg^2+^ ions to enter the aragonite lattice is minimal. As for the inorganically-precipitated aragonite, the energy used for the Mg^2+^ ion incorporation into aragonite was up to 57.37 kcal/mol, much higher than those energy values mentioned above. Thus, the above bio-molecules easily promote the incorporation of Mg^2+^ ions into aragonite due to the decreasing energy, similar to the role played by biocatalysts.

### 3.10. Dolomitization of biotic aragonite

**FIGURE 12:**
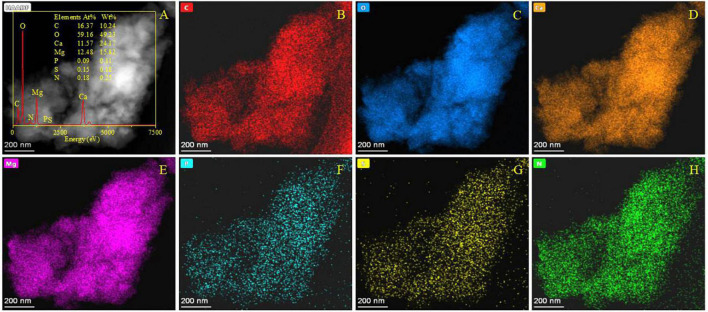
HRTEM, EDS, and elemental analyses of minerals after the dolomitization of bio-aragonite. **(A)** The morphology of minerals in a bright field, the inset showing the EDS result; **(B–H)** indicating the elemental mapping of C, O, Ca, Mg, P, S, and N.

**FIGURE 13:**
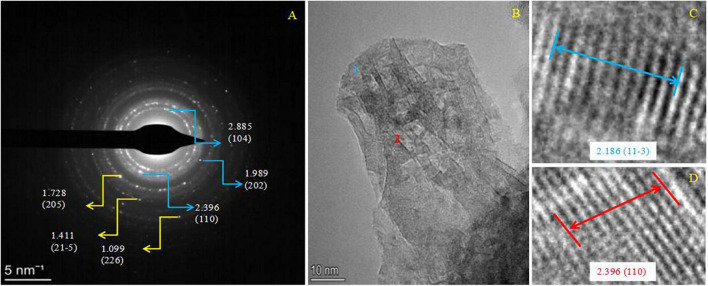
SAED and HRTEM images of minerals after teh dolomitization of bio-aragonite. **(A)** SAED image; **(B,D)** HRTEM images, and **(C,D)** are the interplanar spacing and the hkl indice of the positions 1 and 2 marked in panel **(B)**. PDF card used: dolomite PDF#74-1687.

The minerals derived from the dolomitization of aragonite were further analyzed by SEM accompanied with EDS. As shown in Supplementary Figure 14A, the EDS results show that only a small amount of Mg was detected from the area marked by a red square (Supplementary Figure 14A). However, in the area marked by a yellow circle, a number of rhombic minerals are growing on the surface of biotic aragonite (Supplementary Figure 14B). These rhombic minerals were further analyzed by EDS and the results show that the Mg/Ca atomic ratio reached 0.7. This result suggests these rhombic minerals may be a form of dolomite. When the amount of Mg^2+^ ions incorporated into bio-aragonite increased, the atom ratio of Mg:Ca also increased. Therefore, dolomite may be harvested after a period of dolomitization of biotic aragonite. These minerals derived from the dolomitization of aragonite were further analyzed by HRTEM, EDS, Mapping, and SAED. It can be seen from Figure 12A that the weight percent of elements P, S and N was 0.11, 0.18, and 0.25, suggesting the minerals being analyzed were indeed biotic; besides these three elements, the atom ratio of Mg:Ca was 12.48:11.57, close to 1, indicating that maybe the bio-aragonite has been dolomitized. The elemental mapping results also show that a certain amount of Mg (Figure 12E) is present in addition to C, O, Ca, P, S, and N (Figures 12B–D, F–H). The organic elements P, S, and N (Figures 12F–H) are evenly distributed in the mineral, in line with the biogenesis of aragonite. To further prove the occurrence of dolomitization of biotic aragonite, SAED and HRTEM analyses were performed. It can be seen from Figure 13A that the calculated interplanar spacings *d* (Å) are 2.885, 1.989, 2.396, 1.728, 1.411, and 1.099, well in line with dolomite (PDF#74-1687) *hkl* planes (104), (202), (110), (205), (215), and (226). Two places were selected (Figure 13B) and further analyzed with HRTEM, and the results show that the calculated interplanar spacing *d* (Å) are 2.186 and 2.396, corresponding well to dolomite (PDF#74-1687) *hkl* planes (11-3) and (110) (Figures 13C, D). The above results show that the dolomitization of biotic aragonite has taken place in the presence of bacteria.

## 4. Discussion

### 4.1. Three nucleation sites of prokaryotic cells: EPS, cell wall, and periplasmic space

In recent years, there have been great efforts to determine the roles of the EPS around a cell in biomineralization processes. It has been reported that NH_3_ and CA released by microbial metabolism will create alkaline conditions for the precipitation of carbonate minerals (Zhuang et al., 2018; Guo et al., 2021). The EPS containing different kinds of VOC in this study are negatively charged in these alkaline conditions, facilitating the adsorption of Ca^2+^ and Mg^2+^ ions which could promote the crystal nucleation (Dong et al., 2000; Braissant et al., 2007; Wacey et al., 2007; Deng et al., 2010; Balci et al., 2018; Picard et al., 2018). The nucleation sites of bacteria Y_1_ were clearly the EPS (Figure 5A). Besides the EPS, there is another area that is less obvious, and that is the gap between the cell wall and the membrane (Supplementary Figure 10C). This location is known in biology as periplasmic space. *B. licheniformis* Y_1_ is a gram-positive bacterium, meaning that the cell wall will contain a large amount of peptidoglycan as well as teichoic acid (Brown et al., 2013). The cell wall is thick and the peptidoglycan network molecules form a permeability barrier. When the cells were decolorized by ethanol, the peptidoglycan would be dehydrated and the pore barrier shrinks, thus retaining the crystal violet–iodine complex in the cell wall. Thus, after staining, a gram-positive bacterium is purple. This is the mechanism of Gram staining. Arising from this mechanism, it is known that ions such as Ca^2+^ and Mg^2+^ ions can enter and even cross the permeability barrier formed by peptidoglycan network, due to the fact that much larger molecules, like the crystal violet–iodine complex, can enter into the cell wall in the process of staining, let alone the fact that Ca^2+^ and Mg^2+^ ions have smaller sizes. The Ca^2+^ and Mg^2+^ ions can cross the cell wall and reach the periplasmic space. Therefore, we can observe the amorphous minerals within the cell wall (Supplementary Figure 10B) and in the periplasmic space (Supplementary Figure 10C). As for the nucleation mechanism of these minerals in the cell wall and periplasmic space, it could be that CA released from inside the cell is present in the periplasmic space and cell wall since this is the only way that CA can get out of the cell. CA also contributes to the hydration of carbon dioxide and releases CO_3_^2–^ and HCO_3_^–^ in the alkaline conditions of the cell wall and in the periplasmic space. Thus, supersaturation of minerals can be achieved and then these minerals could be nucleated in these sites. If method could devised to measure the concentrations of Ca^2+^, Mg^2+^, CO_3_^2–^ and HCO_3_^–^ ions and the CA activities within the cell wall and periplasmic space, it would help reveal the mechanism of mineral mineralization in these location. This work will be further explored in the future.

In the experiments of Deng et al. (2010) to induce dolomite precipitation by sulfate reducing bacteria (SRB) and halophilic bacteria, no dolomite was precipitated on the surface of heat-killed cells which lacked EPS, whereas some dolomite crystals were formed within the EPS of living cells. Their results suggested that the EPS could serve as nucleation sites but only when the bacteria were alive. Biomineralization occurs widely in nature, and has been explored in numerous lab experiments. Bio-minerals and the mineralized outer surfaces of bacterial cells formed within microbial mats are found in Bahamas (Diaz et al., 2017) and Qatar (Perri et al., 2018). As for their formation, it has been shown that EPS can accumulate cations through its functional groups and provide nucleation sites for mineral precipitation (Ueshima and Tazaki, 2001; Dupraz and Visscher, 2005; Perri et al., 2018). The bacterial mineralized shells (Supplementary Figure 9C) in this study demonstrate this point. In particular, Perri et al. (2018) and others have suggested that the degradation of EPS by microbes also increases alkalinity to facilitate biomineral precipitation. In the biomineralization process, the bacterial concentration varies with nutrient availability. When nutrients are abundant, the growth ratio is greater than the death ratio, and the concentration of bacteria increases. But when the nutrients are used up, in the case of *Bacillus*, bacteria enter a spore state, with a stable bacterial concentration. In the whole process of bacterial growth, there will always be dead bacteria, and these dead bacteria will decompose and release organic matter, which can be used as the nutrients by living bacteria. The nitrogen-containing organic matter can be metabolized into ammonia gas by living bacteria, which would also further increase the pH value. The results obtained in both the laboratory and field reveal the important role played by EPS in the bio-precipitation process.

The nucleation mechanism of minerals within EPS is related to the negatively-charged functional groups acting as nucleation sites (Szcześ et al., 2016; Han et al., 2018, 2019; Zhuang et al., 2018). The FTIR results (Figure 4) and the amino acid composition of the EPS (Figure 7A) as determined in this study also confirm this process. The negatively-charged functional groups are capable of trapping cations such as Ca^2+^ and Mg^2+^ ions within the EPS to create a supersaturated microenvironment for bio-mineral nucleation and growth. According to the results of amino acid analyses (Figure 7), the most abundant amino acid in the EPS (Figure 7A) and within the biotic aragonite (Figure 7B) is Gly. It has been reported that Gly possesses strong hydrophobic properties due to the presence of two hydrogen atoms in its structure, which likely protect cells by inhibiting the entry of Mg^2+^ ions (Han et al., 2018). Therefore, in this study, *B. licheniformis* Y_1_ needs this Gly to survive in the medium or natural environment with higher and higher Mg^2+^ ion concentrations. In addition to Gly, there are a large number of other amino acids present in the EPS and biotic aragonite, including Glu, Asp, and Ala (Figure 7). The isoelectric points (PIs) of these four amino acids are 5.97, 3.22, 2.77, and 6.00, respectively, indicating that these amino acids are negatively charged in the experimental group because their PIs are much less than pH 8.8. Compared to Gly and Ala, Glu and Asp carry more negative charges due to their two carboxyl groups. In this study, the contents of Glu and Asp are higher both in EPS and biotic aragonite (Figure 7). The presence of these two particular amino acids in the EPS is beneficial to the adsorption of Ca^2+^ and Mg^2+^ ions since they can be deprotonated to become negatively-charged molecules in the alkaline environment. Amino acids such as Glu and Asp play important roles not only in the adsorption of Ca^2+^ and Mg^2+^ ions in the EPS but also in the nucleation of the biotic carbonate minerals themselves once supersaturation is reached. The red fluorescence emitted from the biotic minerals labeled by CY5 NHS ester (inset in Figure 7B), suggests that amino acids (proteins) are incorporated into the biotic aragonite during their nucleation and growth. This is also consistent with the FTIR results that reveal the existence of secondary protein structures (Table 1). In addition to amino acids, the EPS also contain HA, octadecanoic acid and other organic acids (Figure 8A) which also play the same roles in promoting the precipitation of biotic aragonite as mentioned above. These organic acids can also be deprotonated in an alkaline environment and then they carry negative charges to trap Ca^2+^ and Mg^2+^ ions and serve as templates to facilitate the precipitation of aragonite. This is the main reason that these organic acids are present inside the minerals (Figure 8C).

### 4.2. The Mg^2+^ content of biotic aragonite

It has been reported that a higher energy is required to replace Ca^2+^ ions with Mg^2+^ ions in aragonite compared to the case with calcite due to the nine Ca-O bonds within the aragonite crystal (Zhou et al., 2004; Sun et al., 2015). In recent years, the incorporation of Mg^2+^ ions into biogenic aragonite has been researched (Dietzel et al., 2004; Foster et al., 2008; Holcomb et al., 2009; Yao et al., 2019). In general, there is only a minor content of Mg^2+^ in abiotic/inorganically-precipitated aragonite (Yao et al., 2019). Such aragonite analyzed here by AAS contains far less Mg^2+^ than that in the biotic and organically-precipitated aragonite (Figure 9 and Table 2). In this study, the Mg^2+^ content in biotic aragonite induced by *B. licheniformis* Y_1_ increases significantly compared to the inorganically-precipitated aragonite (Figure 9 and Table 2), suggesting that the presence of *B. licheniformis* Y_1_ is strongly facilitating the incorporation of Mg^2+^ into biotic aragonite. From the decreasing interplanar spacing of the aragonite *hkl* plane (111), it can also be concluded that the incorporation of Mg^2+^ into biotic aragonite increased, consistent with the result of calculating the Mg^2+^ content per gram of aragonite. From the inorganically-precipitated aragonite through the organically-precipitated aragonite mediated by Glu to the aragonite induced by bacteria, the interplanar spacing *d* values of the *hkl* planes (111), (121), (102), and (112) decrease in sequence (Figure 10), also suggesting that incorporation of Mg^2+^ into the three kinds of aragonite increased in sequence. However, the micro-environment of Mg^2+^ present in biotic aragonite is still uncertain. The results of X-ray absorption near-edge structure spectroscopy (XANES) indicate that the Mg^2+^ present in biotic aragonite is associated with organics (Finch and Allison, 2008; Yao et al., 2019). However, based on the results of Rayleigh-based multi-element thermometer analysis, Gaetani et al. (2011) suggested that in biotic aragonite, Mg^2+^ was directly substituting for Ca^2+^ in the aragonite crysta; this has also been proved by the measurements of Mg isotope fraction factors (Wang Z. et al., 2013; Saenger and Wang, 2014). Furthermore, the results of DFT calculations demonstrate that Mg^2+^ substituted Ca^2+^ in abiotic aragonite in a five-fold coordinated structure (Son et al., 2020). In this study, the results of HRTEM and SAED showed that with the increase of Mg^2+^ ion content in aragonite, the *d* values of the *hkl* planes (111), (121), (102), and (112) decrease (Figure 10), which likely indicates that the incorporated Mg^2+^ ions in biotic and organically-precipitated aragonite lattices replaces Ca^2+^ ions. It is well known that the ionic radius of Mg^2+^ (*r* = 0.86 Å) is smaller than that of Ca^2+^ (*r* = 1.14 Å) (Shannon, 1976), accounting for the decrease in *d* values of crystal planes when Mg^2+^ substitutes for Ca^2+^ (Zhang et al., 2010).

In addition to the precipitation of biotic aragonite being affected by bacteria, the Mg^2+^ ions are also incorporated into biotic aragonite much more easily due to the presence of the biomolecules. In view of its orthorhombic structure, Mg^2+^ is difficult to be incorporated into aragonite (Yao et al., 2019). Yao et al. (2019) proved that the Mg^2+^ content really increased in their aragonite precipitates meditated by SA and CC. In this study, the detailed mechanism of Mg^2+^ incorporation into biotic and organically-precipitated aragonite is further explored. The content of Mg^2+^ in aragonite increases significantly with increasing amounts of HA, Glu, Gly and Ala (Figure 9 and Supplementary Table 8), indicating that higher concentration of biomolecules can promote the incorporation of Mg^2+^ ions into aragonite much more easily than lower concentration when using the same biomolecule. In view of the slight solubility of HA, we only prepared the saturated solution of HA. The Mg^2+^ content in aragonite precipitates mediated by HA is higher than that in the aragonite formed in the solution with a lower concentration of Gly and Ala (0.05 g/L) (Figure 9). There is another conclusion to be drawn from Figure 9, namely, at the same concentration, the abilities of different biomolecules are also different in the Mg^2+^ ion incorporation into aragonite, Glu > Gly > Ala (Supplementary Table 8). In order to study the reason behind this result, the energy needed for the incorporation of Mg^2+^ ions into aragonite was calculated. After the DFT calculation, the energy needed for the Mg^2+^ incorporation into aragonite mediated by Glu, Gly and Ala was from low to high in a sequence (Figure 11). The incorporation of Mg^2+^ ions into the lattice of aragonite requires energy. The lower the energy required, the easier it is for Mg^2+^ ions to enter the lattice of aragonite; the higher the energy required, the less likely it is for Mg^2+^ ions to enter the lattice of aragonite. In this study, the energies used for Mg^2+^ incorporation into aragonite mediated by biomolecules are much lower than those formed in the inorganic solution after the DFT calculation, indicating that the presence of biomolecules, especially the biomolecules in EPS, can promote the Mg^2+^ ion incorporation into biotic aragonite more easily than the inorganiclly-precipitated aragonite. It is the main reason for the higher Mg^2+^ ion contents of biotic aragonite than organically-precipitated aragonite.

### 4.3. Implications for the dolomitization of biotic aragonite

The “dolomite enigma” has been a mystery for two centuries at least; compared to the common occurrence in pre-Holocene strata, dolomite is relatively rare in modern marine sediments (de Dolomieu, 1791; Gregg et al., 2015; Qiu et al., 2017; Zheng et al., 2021). After various experiments over many decades, a most popular way to synthesize dolomite in laboratory experiments under earth surface conditions has been to utilize microbes (Sánchez-Román et al., 2009, 2011; Qiu et al., 2019; Liu et al., 2020; Han et al., 2022). The replacement of Ca^2+^ ions by Mg^2+^ ions in carbonate minerals has been regarded as the major reason for the formation of dolomite, but this has rarely been reported from laboratory experiments at Earth surface conditions (Kaczmarek and Thornton, 2017; Zheng et al., 2021). The equation:

$$2\,C\,a\,C\,O_{3}\,\left(s\right)+M\,g^{2+}\,\left(a\,q\right)=C\,a\,M\,g\,{\left(C\,O_{3}\right)}_{2}\,\left(s\right)+C\,a^{2+}\,\left(a\,q\right)$$

represents the dolomitization of aragonite or calcite. Compared to calcite, the dolomitization of aragonite is more difficult due to its orthorhombic structure. Wang et al. (2022) used calcite as a template to study the dolomitization of calcite over a period of 3 months in a fluid system with bacteria and various Mg^2+^ concentrations and high salinity. Their results suggested that the calcite transformed to dolomite gradually under the influence of extreme halophilic bacteria. They suggested that the dolomitization of calcite occurred through the dissolution of the calcite surface. Compared to calcite, there are few reports of the dolomitization of aragonite. In a low temperature experiment, Chazen and Ehrlich (1973) used submicron aragonite crystals as seeds to study dolomitization in a solution of artificial seawater (NaCl 26.518 g/kg, MgCl_2_ 4.447 g/kg, MgSO_4_ 3.305 g/kg, CaCl_2_ 1.141 g/kg, KCl 0.725 g/kg, NaHCO_3_ 0.202 g/kg, and NaBr 0.083 g/kg). After the solution evaporated to nearly dry (salinity arpprox. 7 times seawater), they detected dolomite in the precipitates. Zempolich and Baker (1993) used aragonite ooids as seeds to study the dolomitization of aragonite in laboratory experiments, but at 200°C. They obtained ordered dolomite using a solution containing 0.5 mol/L Mg^2+^ and 0.4 mol/L Ca^2+^ within 180 h, and suggested an intermediate form of Ca-Mg carbonate formed first. In natural environments, there are many factors which could affect the dolomitization of aragonite, including bacteria which are ubiquitous in most environments. In this study, we used biotic aragonite as the template to study dolomitization of aragonite in a Mg^2+^-rich solution for several months. Our experimental results proved that during the culture period, the acidic biomolecules in the EPS of *B. licheniformis* Y_1_ facilitated the incorporation of Mg^2+^ ions into the biotic aragonite lattice. With the increase in Mg^2+^ content of the aragonite, more Ca^2+^ ions there would have been replaced by Mg^2+^ ions, so that the surface of the biotic aragonite became dolomitized. It is also possible that Ca^2+^ ions released from the surface of the biotic aragonite reacted with Mg^2+^ ions and CO_3_^2–^ ions in the ambient solution to form dolomite directly under the action of different biomolecules. These are just two hypotheses that have been extrapolated from our experiments so far. The above conclusions have confirmed that the action of microorganisms is more conducive to the incorporation of Mg^2+^ ions into the biotic aragonite. Meanwhile, the energy required for the incorporation of Mg^2+^ ions into aragonite under the action of various organic molecules in EPS is much less. When the amount of incorporated Mg^2+^ ions is sufficient, will the dolomitization of aragonite have occurred? The specific dolomitization process of the biotic aragonite and its mechanisms will be examined in more detail in the future.

## 5. Conclusion

Our experiments show the Mg^2+^ contents in biotic/organically-precipitated aragonite is much higher than those in inorganically-precipitated aragonite. The bacteria *B. licheniformis* Y1 create alkaline conditions leading to the precipitation of aragonite. The EPS, cell wall and periplasmic space provide templates for the nucleation and growth of biotic minerals. An important role of biomolecules for the incorporation of Mg^2+^ into biotic aragonite is demonstrated. Acidic biomolecules (like HA) produced by bacteria during the biomineralization process can significantly reduce the energy for the incorporation of Mg^2+^ into aragonite. Our experiments also suggest that in a bacterial system with Mg^2+^, dolomite is able to grow on the biotic aragonite surface with the increase in culture time. These results may provide some insights towards a better understanding of the dolomitization of aragonite.

## Data availability statement

The original contributions presented in this study are included in the article/Supplementary material, further inquiries can be directed to the corresponding author.

## Author contributions

ZH wrote and revised the manuscript. RM performed the experiments and analyzed data. HZ responded to reviewers and revised the manuscript. XG calculated the DFT. YZ analyzed the data. YH helped to perform the experiments. FL calculated and simulated with the software of Material Studio. MT contributed to the revision and grammar. JD calculated the interplanar spacing of minerals. HY contributed to the conception of the study and revision of manuscript. All authors contributed to the article and approved the submitted version.

## Supplementary material

Supplementary Figure 1 Phylogenetic tree constructed with neighbor-joining method based on a sequence alignment of bacterial 16S rRNA genes.

Supplementary Figure 2 SEM image of *B. licheniformis* Y_1_ **(A)** and the qualitative experiment of ammonia **(B,C)**.

Supplementary Figure 3 The growth curve of *B. licheniformis* Y_1_ and pH changes.

Supplementary Figure 4 Changes of saturation index of calcite in aqueous media during the process of biomineralization.

Supplementary Figure 5 Rietveld refinement of the biotic minerals formed at different Mg/Ca molar ratios.

Supplementary Figure 6 Scanning electron microscope images of the bio-minerals induced by *B. licheniformis* Y_1_ at different Mg/Ca ratios. **(A–C)** Calcite formed at a Mg/Ca molar ratio of 0; **(D–F)** Mg-calcite formed at a Mg/Ca molar ratio of 3; **(G)** Mg-calcite formed at a Mg/Ca molar ratio of 6; and **(H–I)** Mg-calcite formed at a Mg/Ca molar ratio of 9.

Supplementary Figure 7 The results of EDS analyses of biominerals induced by *B. licheniformis* Y_1_ at different Mg/Ca molar ratios.

Supplementary Figure 8 FTIR spectra of the biotic aragonite after 25 days of cultivation.

Supplementary Figure 9 SEM images of *B. licheniformis* Y_1_ cells.

Supplementary Figure 10 Ultrathin slices of *B. licheniformis* Y_1_ cells analyzed by HRTEM and SAED. No biomineralization **(A)**, the biomineralization occurring in/on EPS **(B–D)**, and the intracellular biomineralization. Inset in panel **(D)**, SAED spectra of the nanometer-scaled dark spherical area in figure **D**.

Supplementary Figure 11 HRTEM and SAED images of ultrathin slices of *B. licheniformis* Y_1_ cells. The insets in panels **(E,F)** are the SAED images of bio-minerals on the cell surface marked with red squares in panels **(B,D)**, respectively. The original figures of the insets are in Supplementary Figure 12. Wt and At% shown in EDS results **(E,F)** means the weight ratio and atomic ratio of the elements contained in the bio-minerals marked with red squares **(B,D)**, respectively.

Supplementary Figure 12 SAED analyses of the nanometer-scaled minerals on the cell surface. The original figures of insets in Supplementary Figure 11.

Supplementary Figure 13 SEM images of aragonite precipitates mediated by various biomolecules with different concentrations. The biomolecules used in this experiment: **(A)** without any biomolecules; **(B)** saturated HA solution; **(C–E)** amino acid Ala; **(F–H)** amino acid Gly; and **(I–K)** amino acid Glu.

Supplementary Figure 14 SEM and EDS results of minerals derived from the dolomitization of aragonite. **(A)** Minerals obtained from the media used for the dolomitization of aragonite after culturing for two month; **(B)** the enlarged image of the area marked by yellow circle in panel **(A)**.
