# Supplementary material for: The incorporation of Mg2+ ions into aragonite during biomineralization: Implications for the dolomitization of aragonite
Source: Front Microbiol. 2023 Jan 26;14:1078430. doi: 10.3389/fmicb.2023.1078430 (PMC9909399; doi:10.3389/fmicb.2023.1078430)
Supplement: Supplementary file 1 [file Data_Sheet_1.DOCX]

**Table S1** The significance of difference in pH at different Mg/Ca ratios with incubation time.

Note: **indicating the extremely significant difference (P<0.01); *indicating the significant difference (0.01<P<0.05); - indicating no comparision.

**Table S2** The significance of difference in HCO_3_^-^ concentration at different Mg/Ca ratios with incubation time.

Note: **indicating the extremely significant difference (P<0.01); *indicating the significant difference (0.01<P<0.05); - indicating no comparision.

**Table S3** The significance of difference in CO_3_^2-^ concentration at different Mg/Ca ratios with incubation time.

Note: **indicating the extremely significant difference (P<0.01); *indicating the significant difference (0.01<P<0.05); - indicating no comparision.

**Table S4** The significance of difference in Ca^2+^ concentration at different Mg/Ca ratios with incubation time.

Note: **indicating the extremely significant difference (P<0.01); *indicating the significant difference (0.01<P<0.05); - indicating no comparision.

**Table S5** The significance of difference in Mg^2+^ concentration at different Mg/Ca ratios with incubation time.

Note: **indicating the extremely significant difference (P<0.01); *indicating the significant difference (0.01<P<0.05); - indicating no comparision.

**Table S6** The significance of difference in saturation index at different Mg/Ca ratios with incubation time.

Note: **indicating the extremely significant difference (P<0.01); *indicating the significant difference (0.01<P<0.05); - indicating no comparision.

**Table S7** The interplanar spacing (*d*, Å) of aragonite *hkl* plane (111) (PDF#71-2396) with increasing Mg^2+^ ion concentration.

| Mg/Ca ratio | Interplanar spacing of aragonite *hkl* plane (111) (Å) | |
| --- | --- | --- |
|  | 14^th^ day | 25^th^ day |
| 6 | 3.427 | 3.421 |
| 9 | 3.419 | 3.416 |
| 12 | 3.411 | 3.404 |

**Table S8** The significance of difference in Mg^2+^ content in aragonite mediated by biomolecules.

**
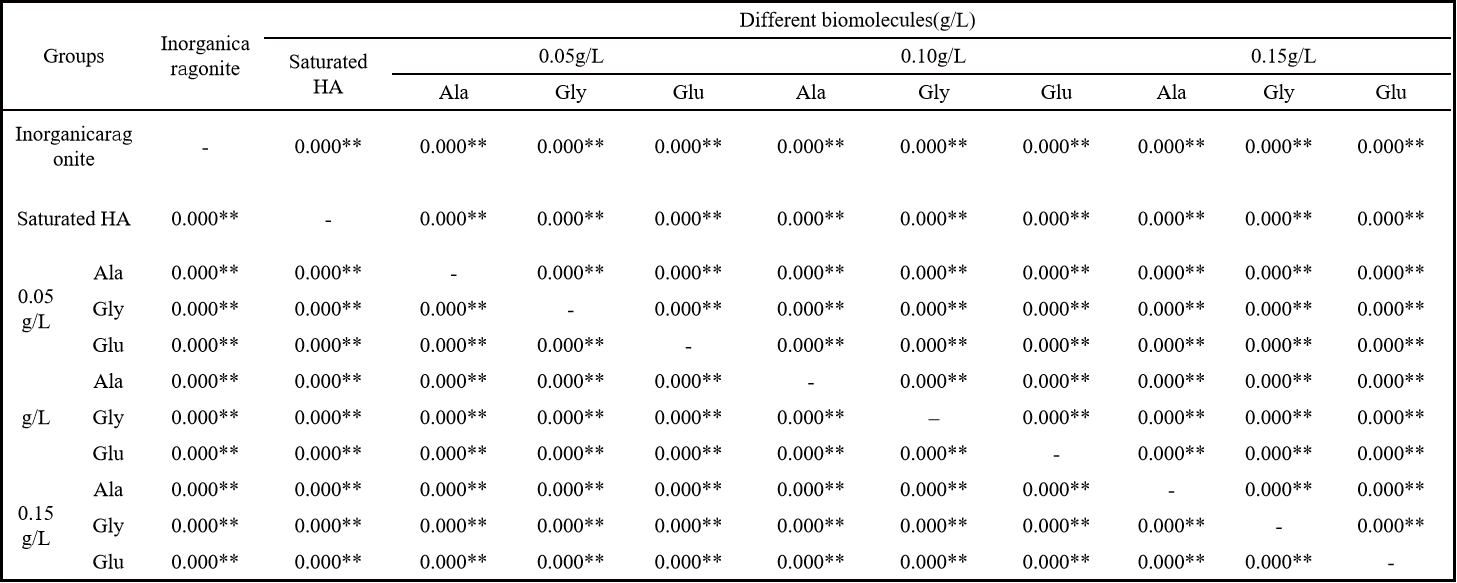
**

Note: ** indicating the extremely significant difference (P<0.01); * indicating the significant difference (0.01<P<0.05); - indicating no comparision.
