# Supplementary figures and images for: The incorporation of Mg2+ ions into aragonite during biomineralization: Implications for the dolomitization of aragonite
Source: Front Microbiol. 2023 Jan 26;14:1078430. doi: 10.3389/fmicb.2023.1078430 (PMC9909399; doi:10.3389/fmicb.2023.1078430)

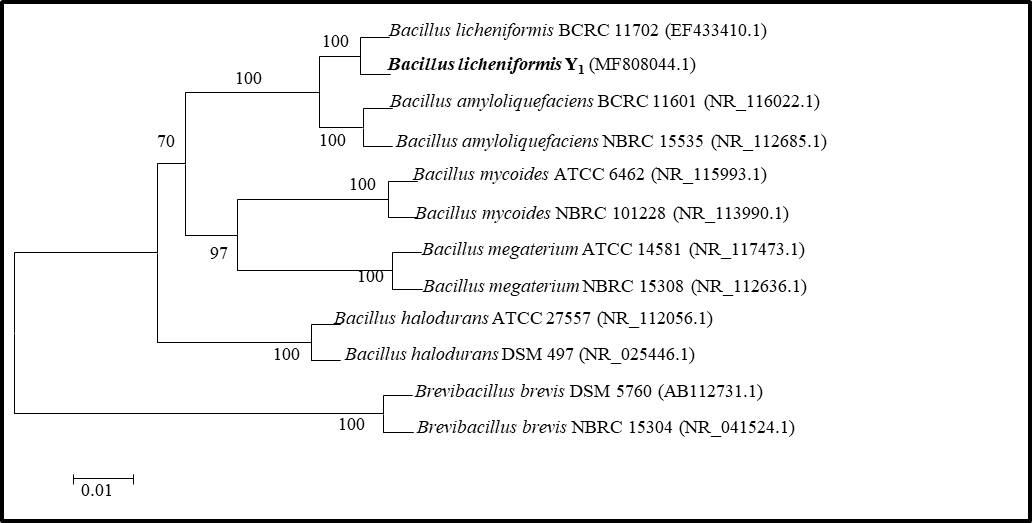

Supplement: Supplementary Figure 1 — Phylogenetic tree constructed with neighbor-joining method based on a sequence alignment of bacterial 16S rRNA genes. [file Image_1.JPEG]

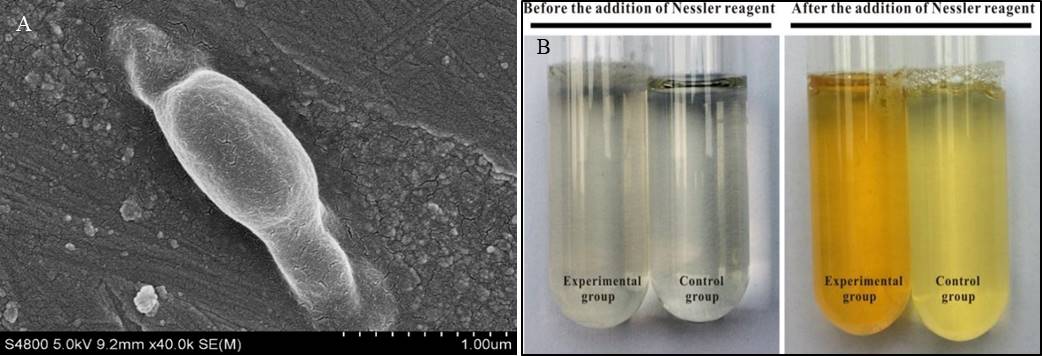

Supplement: Supplementary Figure 2 — SEM image of B. licheniformis Y1 (A) and the qualitative experiment of ammonia (B,C). [file Image_2.JPEG]

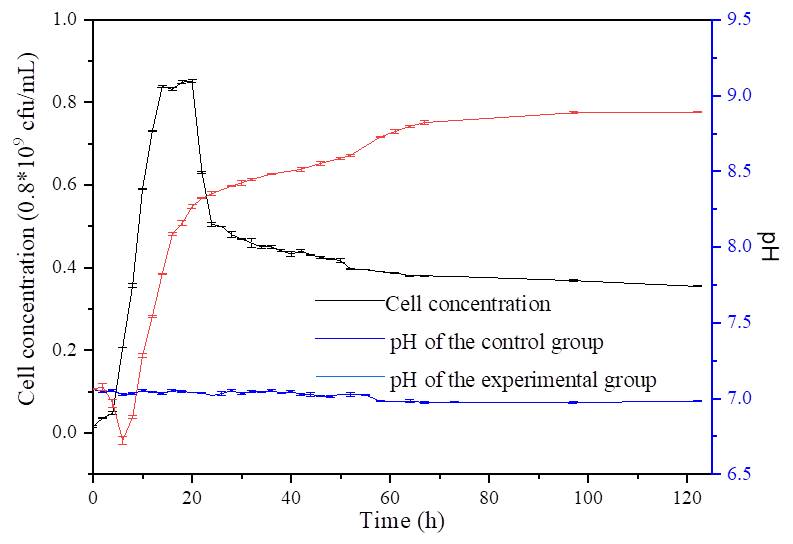

Supplement: Supplementary Figure 3 — The growth curve of B. licheniformis Y1 and pH changes. [file Image_3.JPEG]

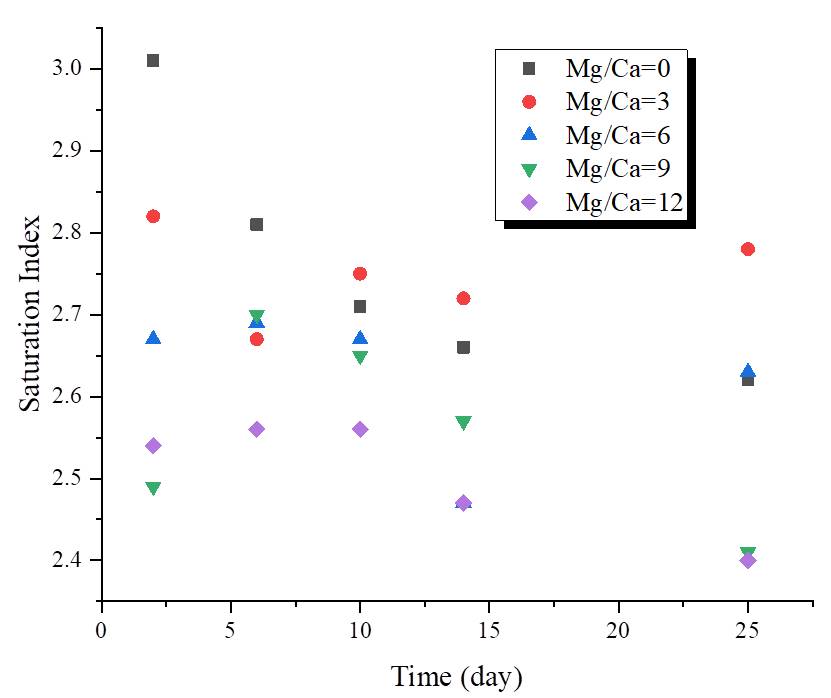

Supplement: Supplementary Figure 4 — Changes of saturation index of calcite in aqueous media during the process of biomineralization. [file Image_4.JPEG]

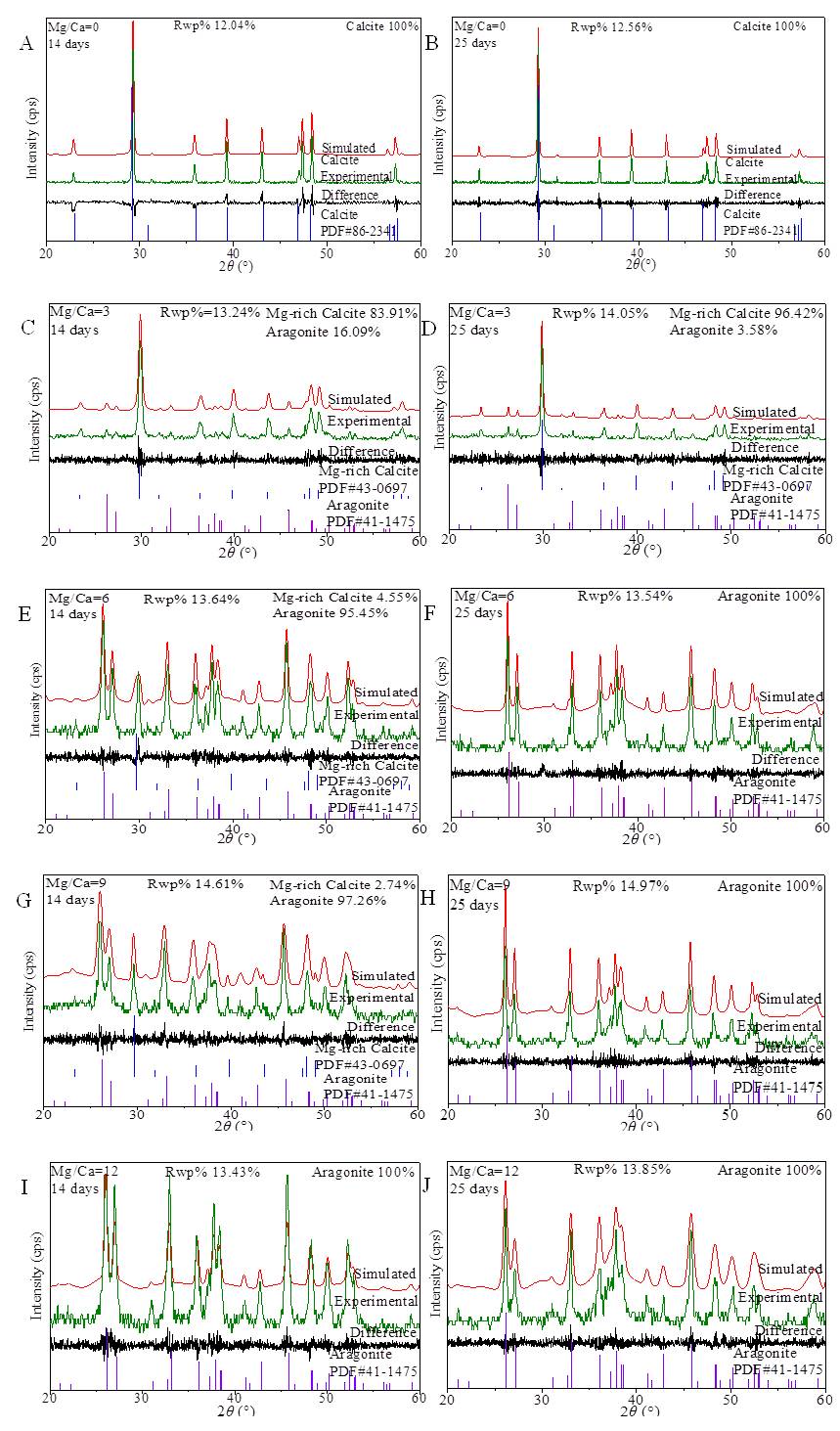

Supplement: Supplementary Figure 5 — Rietveld refinement of the biotic minerals formed at different Mg/Ca molar ratios. [file Image_5.JPEG]

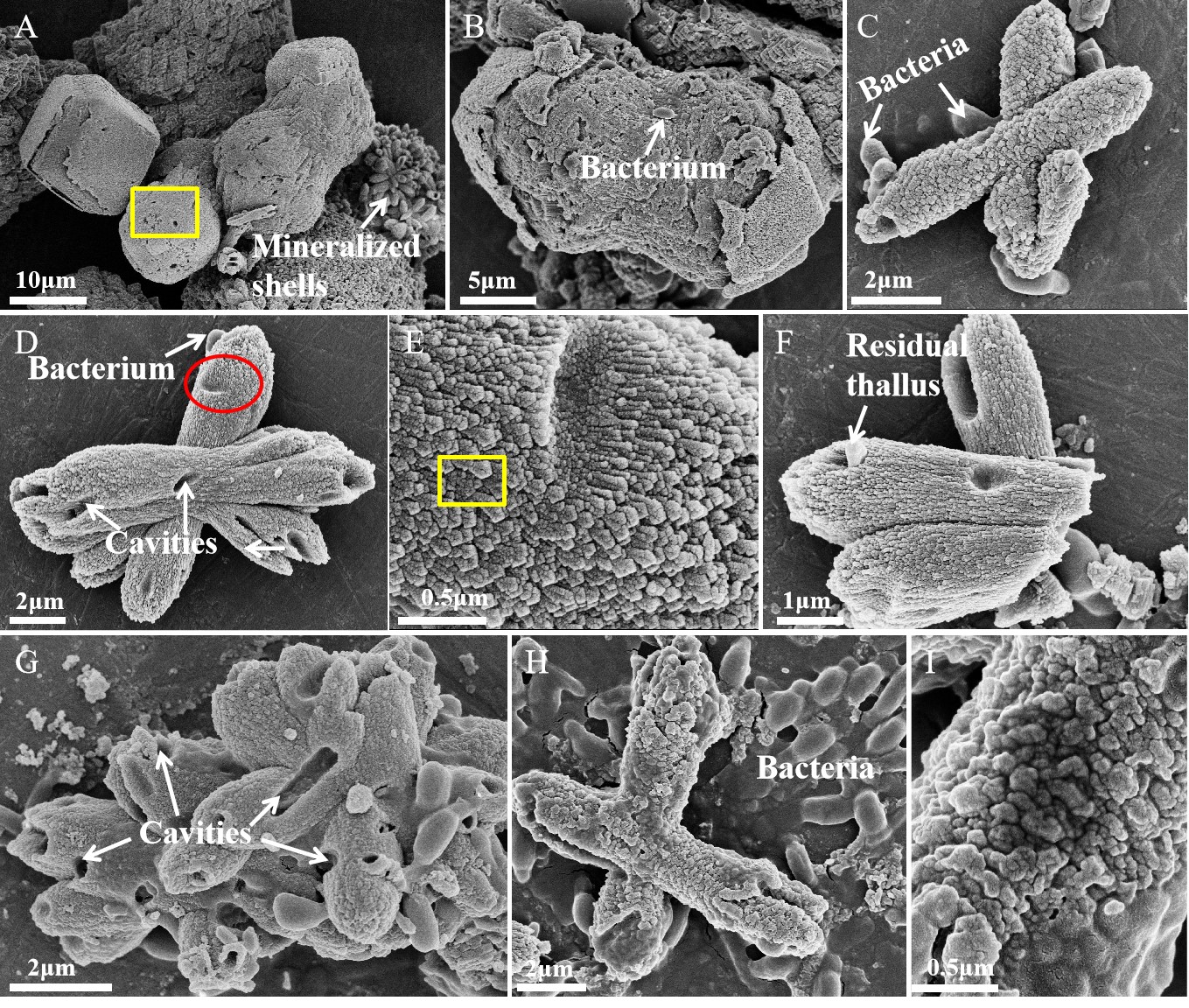

Supplement: Supplementary Figure 6 — Scanning electron microscope images of the bio-minerals induced by B. licheniformis Y1 at different Mg/Ca ratios. (A–C) Calcite formed at a Mg/Ca molar ratio of 0; (D–F) Mg-calcite formed at a Mg/Ca molar ratio of 3; (G) Mg-calcite formed at a Mg/Ca molar ratio of 6; and (H–I) Mg-calcite formed at a Mg/Ca molar ratio of 9. [file Image_6.JPEG]

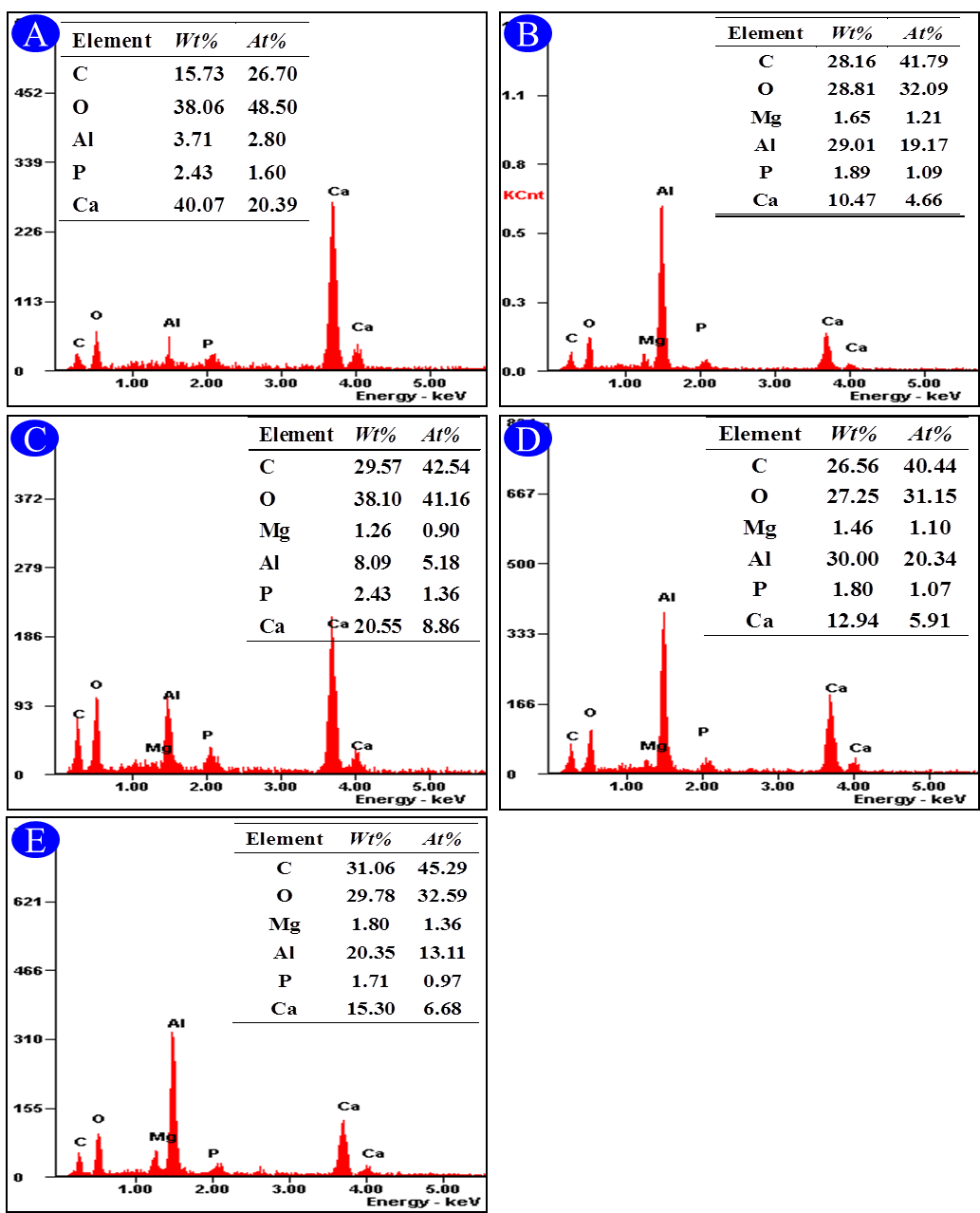

Supplement: Supplementary Figure 7 — The results of EDS analyses of biominerals induced by B. licheniformis Y1 at different Mg/Ca molar ratios. [file Image_7.PNG]

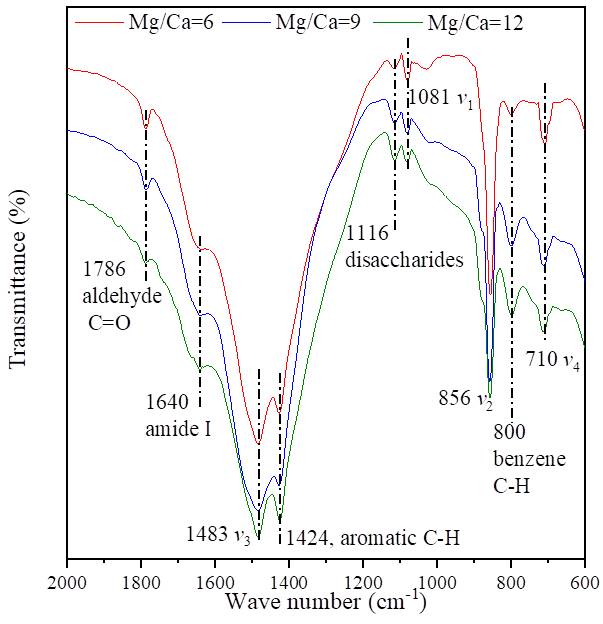

Supplement: Supplementary Figure 8 — FTIR spectra of the biotic aragonite after 25 days of cultivation. [file Image_8.JPEG]

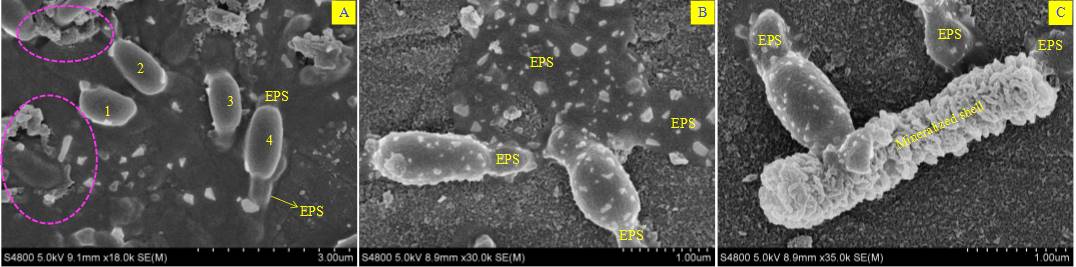

Supplement: Supplementary Figure 9 — SEM images of B. licheniformis Y1 cells. [file Image_9.JPEG]

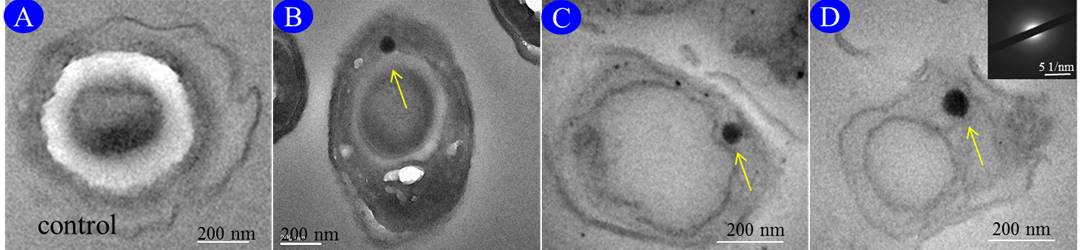

Supplement: Supplementary Figure 10 — Ultrathin slices of B. licheniformis Y1 cells analyzed by HRTEM and SAED. No biomineralization (A), the biomineralization occurring in/on EPS (B–D), and the intracellular biomineralization. Inset in panel (D), SAED spectra of the nanometer-scaled dark spherical area in figure D. [file Image_10.JPEG]

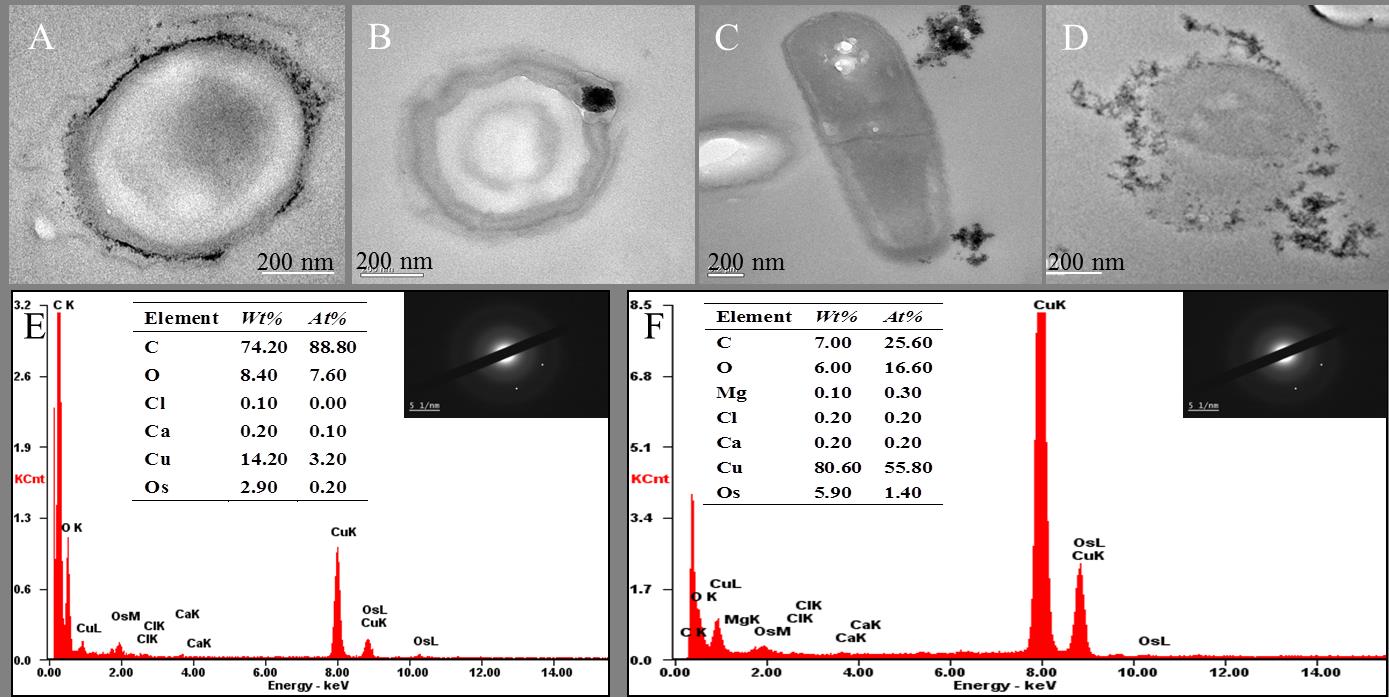

Supplement: Supplementary Figure 11 — HRTEM and SAED images of ultrathin slices of B. licheniformis Y1 cells. The insets in panels (E,F) are the SAED images of bio-minerals on the cell surface marked with red squares in panels (B,D), respectively. The original figures of the insets are in Supplementary Figure 12. Wt and At% shown in EDS results (E,F) means the weight ratio and atomic ratio of the elements contained in the bio-minerals marked with red squares (B,D), respectively. [file Image_11.JPEG]

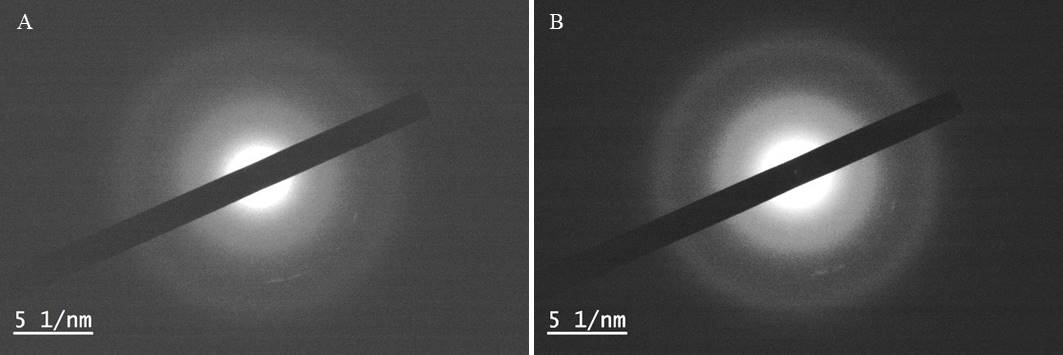

Supplement: Supplementary Figure 12 — SAED analyses of the nanometer-scaled minerals on the cell surface. The original figures of insets in Supplementary Figure 11. [file Image_12.JPEG]

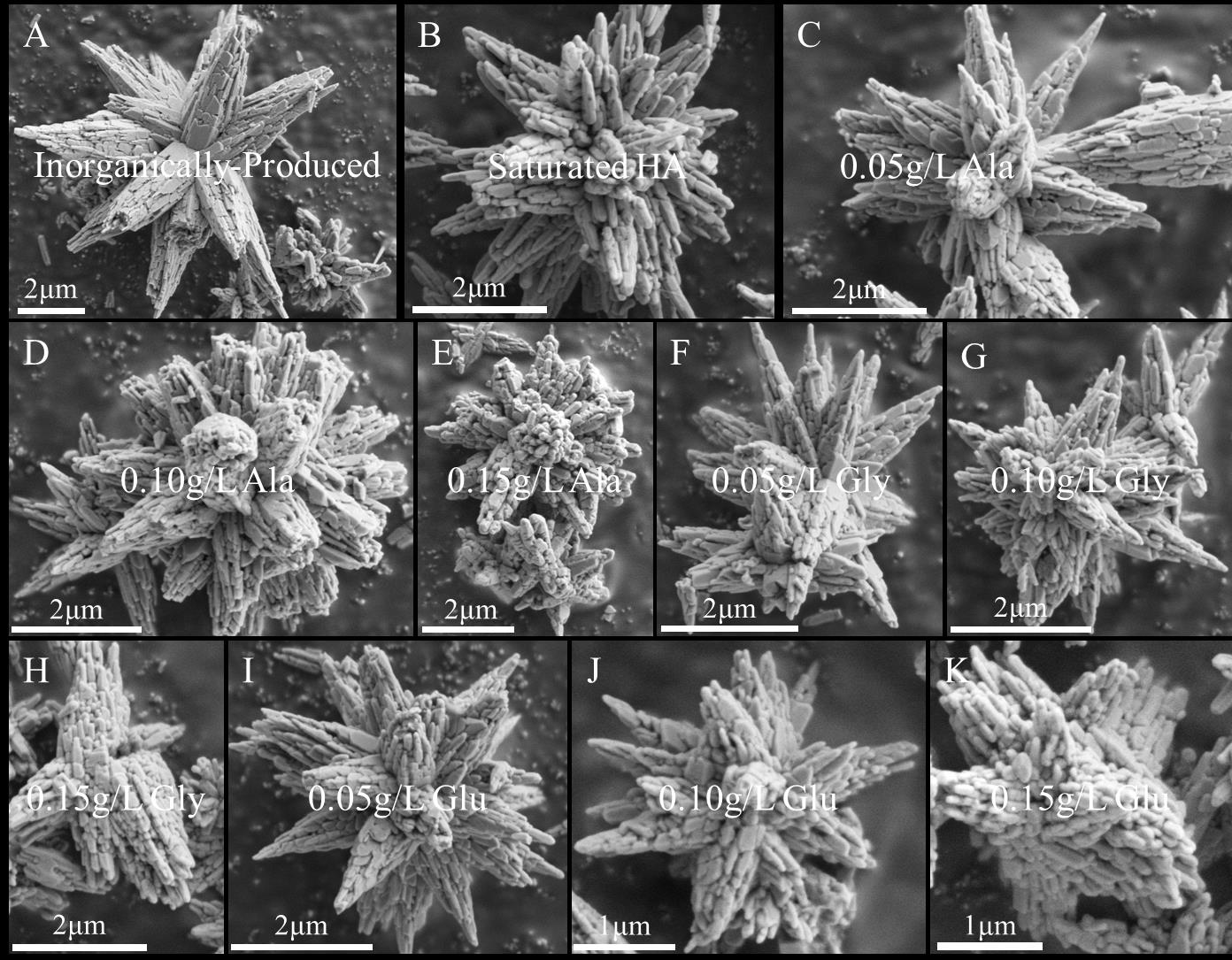

Supplement: Supplementary Figure 13 — SEM images of aragonite precipitates mediated by various biomolecules with different concentrations. The biomolecules used in this experiment: (A) without any biomolecules; (B) saturated HA solution; (C–E) amino acid Ala; (F–H) amino acid Gly; and (I–K) amino acid Glu. [file Image_13.JPEG]

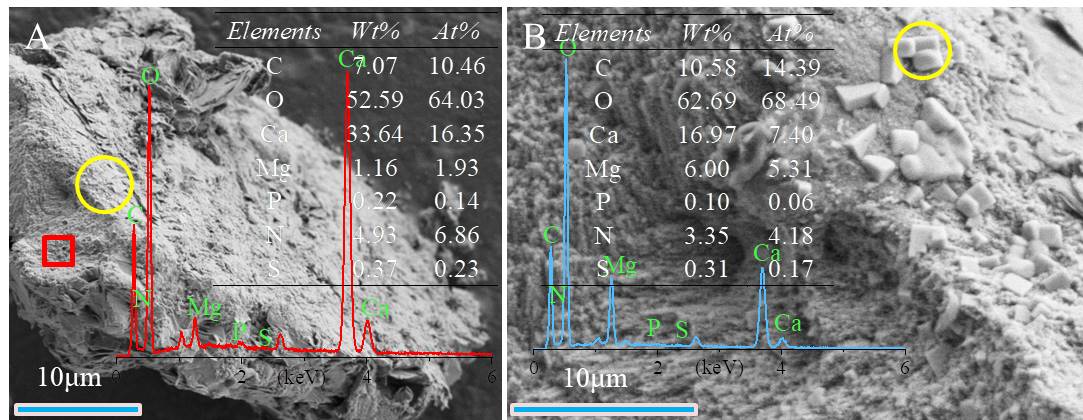

Supplement: Supplementary Figure 14 — SEM and EDS results of minerals derived from the dolomitization of aragonite. (A) Minerals obtained from the media used for the dolomitization of aragonite after culturing for two month; (B) the enlarged image of the area marked by yellow circle in panel (A). [file Image_14.JPEG]
